# The human chromatin remodeling complex p400 restricts HIV-1 transcription in a Tat-dependent manner

**DOI:** 10.1093/nar/gkaf1323

**Published:** 2025-12-18

**Authors:** Chuan Li, Yuan Ma, Luisa P Mori, Huiming Yang, Yi Wang, Thomas T Venables, Ronald Bronson, Ryan R Milione, Mathew E Pipkin, Susana T Valente

**Affiliations:** Department of Immunology and Microbiology, The Herbert Wertheim UF Scripps Institute for Biomedical Innovation & Technology, Jupiter, FL 33458, United States; Department of Immunology and Microbiology, The Herbert Wertheim UF Scripps Institute for Biomedical Innovation & Technology, Jupiter, FL 33458, United States; Department of Immunology and Microbiology, The Herbert Wertheim UF Scripps Institute for Biomedical Innovation & Technology, Jupiter, FL 33458, United States; The Skaggs Graduate School of Chemical and Biological Sciences, The Scripps Research Institute, Jupiter, FL 33458, United States; Department of Immunology and Microbiology, The Herbert Wertheim UF Scripps Institute for Biomedical Innovation & Technology, Jupiter, FL 33458, United States; Department of Immunology and Microbiology, The Herbert Wertheim UF Scripps Institute for Biomedical Innovation & Technology, Jupiter, FL 33458, United States; Department of Immunology and Microbiology, The Herbert Wertheim UF Scripps Institute for Biomedical Innovation & Technology, Jupiter, FL 33458, United States; Department of Immunology and Microbiology, The Herbert Wertheim UF Scripps Institute for Biomedical Innovation & Technology, Jupiter, FL 33458, United States; Department of Immunology and Microbiology, The Herbert Wertheim UF Scripps Institute for Biomedical Innovation & Technology, Jupiter, FL 33458, United States; Department of Immunology and Microbiology, The Herbert Wertheim UF Scripps Institute for Biomedical Innovation & Technology, Jupiter, FL 33458, United States; The Skaggs Graduate School of Chemical and Biological Sciences, The Scripps Research Institute, Jupiter, FL 33458, United States; Department of Immunology and Microbiology, The Herbert Wertheim UF Scripps Institute for Biomedical Innovation & Technology, Jupiter, FL 33458, United States; The Skaggs Graduate School of Chemical and Biological Sciences, The Scripps Research Institute, Jupiter, FL 33458, United States

## Abstract

The chromatin landscape surrounding integrated HIV proviruses critically shapes viral transcription. We systematically examined ATP-dependent chromatin remodeling complexes (SWI/SNF, ISWI, CHD, and INO80) as regulators of HIV expression and identified the p400 complex, a member of the INO80 family, as a potent repressor. Depleting p400 subunits, including the EP400 ATPase and DMAP1, markedly increased HIV transcription and RNAPII elongation at the proviral locus. Mechanistically, EP400 associates with the RNAPII C-terminal domain, while DMAP1 directly engages the viral transactivator Tat, with repression requiring simultaneous interactions among EP400, DMAP1, and Tat. Loss of either EP400 or DMAP1 selectively increased infection and transcription of Tat-competent, but not Tat-deficient, viruses. Although p400 is recruited to active HIV chromatin via RNAPII in a Tat-independent manner, it restrains elongation once Tat accumulates during reactivation. DMAP1 binding to Tat’s basic domain blocks Tat-TAR RNA interaction, thereby limiting p-TEFb-mediated RNAPII Ser2 phosphorylation and elongation. Thus, the p400 complex functions as a host restriction factor that limits Tat-dependent HIV transcription via a Tat-dependent proximal mechanism, highlighting the p400-Tat interface as a potential target for HIV cure strategies.

## Graphical Abstract

**Graphical Abstract:**
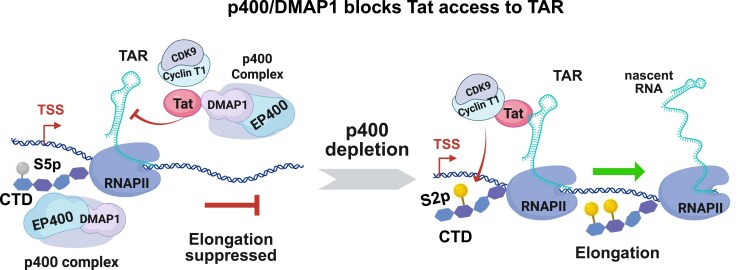

## Introduction

Current antiretroviral therapy (ART) effectively suppresses HIV replication but is not curative; people living with HIV (PLWH) must remain on treatment for life to prevent viral rebound. HIV predominantly persists in a transcriptionally silent form within long-lived memory CD4⁺ T cells, forming a latent reservoir that is the major barrier to cure [1, 2]. Eliminating or permanently silencing this reservoir remains a central challenge in HIV research, underscoring the need for a deeper understanding of the molecular mechanisms governing transcriptional activation and latency maintenance.

The HIV promoter at the 5′ long terminal repeat (5′LTR) is controlled by coordinated spatiotemporal interactions between cellular transcription factors (TFs), chromatin regulators (CRs), and the viral transcriptional transactivator Tat. Tat amplifies transcription by recruiting host elongation machinery, creating a self-amplifying positive-feedback loop [3]. Initially translated from multiply spliced HIV transcripts, Tat accumulates to a threshold that enables efficient recruitment of positive transcription elongation factor b (p-TEFb; composed of CDK9 and Cyclin T1/CCNT1) to the transactivation response (TAR) RNA element of nascent HIV transcripts [4]. This recruitment promotes assembly of the Super Elongation Complex (SEC; ENL, AF9, ELL2, and AFF4) [5, 6]. CDK9 then phosphorylates the *C*-terminal domain (CTD) of RNA polymerase II (RNAPII) at Ser2, as well as the pausing factors DSIF and NELF, thereby releasing RNAPII from promoter-proximal pausing and enabling productive elongation [7]. Structurally, the HIV transcription start site (TSS) is flanked by two nucleosomes, Nuc-0 and Nuc-1, irrespective of the viral sequence or integration site [8, 9]. Tat also recruits histone acetyltransferases and ATP-dependent chromatin remodelers to reposition Nuc-1 into a transcription-permissive configuration [10−16]. Through various mechanisms, Tat can thus upregulate HIV transcription by up to 300% relative to cellular activation alone [17].

Beyond viral factors, the host epigenetic machinery modulates HIV proviral transcription by writing, erasing, and reading histone post-translational modifications and by repositioning nucleosomes via ATP-dependent chromatin remodeling complexes (CRCs) [18]. CRCs are multi-subunit machines built around an SNF2 (sucrose non-fermenting)-type ATPase subunit that hydrolyzes ATP to disrupt histone-DNA contacts, thereby regulating dynamic access to packaged DNA [19]. Based on ATPase domain architecture, the 16 human CRCs are grouped into four families: switch/sucrose non-fermenting (SWI/SNF) family, imitation switch (ISWI) family, chromodomain helicase DNA-binding (CHD) family, and the Inositol auxotrophy 80 (INO80) family, with the INO80-related complexes often mediating histone-variant exchange [20]. To date, HIV transcriptional regulation has been most extensively linked to the SWI/SNF family, particularly BAF and PBAF complexes [21], while CHD1/2, CHD3, and CHD9 have been implicated but lack detailed mechanistic definition [11, 22, 23].

To broaden our understanding of CRC involvement in HIV transcription, we performed an shRNA (short hairpin RNA) screen targeting all 16 SNF2-family ATPase subunits in two HIV latency models (J-Lat 10.6 and Jurkat A2) [24]. This screen identified the p400 complex, an INO80-family remodeler, as a potent repressor of Tat-driven HIV-1 transcription and a key player in maintaining proviral latency. The human p400 complex, also known as NuA4 or the TIP60 complex [25], comprises ∼20 subunits, including the ATPase EP400; scaffold/core subunits (TRRAP, DMAP1, BAF53A, GAS41, Actin); a histone acetyltransferase (HAT) module (EPC1, KAT5, MBTD1, MEAF6, ING3); and additional components (YL1, BRD8, RUVBL1, RUVBL2, MRGX, MRGBP, JAZF1) [26−35]. p400 mediates histone-variant exchange at DNA double-strand breaks, the p21 promoter, and promoters of PPAR-γ target genes, contributing to DNA damage repair, homologous recombination, cell proliferation, senescence, and adipogenesis [36−43]. Its function is context dependent: p400 can activate transcription, e.g. via interactions with adenovirus early region 1A (E1A) and Merkel cell polyomavirus (MCPyV) small T antigen to promote cellular transformation and reprogramming [44−46]; or repress transcription, as seen with its interactions with human papillomavirus (HPV) E2 protein and the hepatitis B virus (HBV) precore/core promoter [47, 48].

In the context of HIV, we found that p400 depletion markedly increased RNAPII occupancy at the proviral locus, with a pronounced rise in Ser2-phosphorylated RNAPII, indicating that p400 restrains transcriptional elongation. Mechanistically, DMAP1 binds Tat directly, while EP400 engages with the RNAPII CTD. Loss- and gain-of-function studies revealed that effective repression of HIV transcription requires concurrent interactions between DMAP1, EP400, and Tat. Using the HIV reporter vector HIV_GKO_ [49] and its Tat-deficient counterpart (HIV_GKO_-ΔTat), we found that during acute infection, knockdown of EP400 or DMAP1 significantly increased the fraction of cells actively transcribing HIV_GKO_, but not HIV_GKO_-ΔTat, establishing p400 as a Tat-dependent transcriptional repressor. Multiple orthogonal approaches demonstrated that p400 binds the basic region of Tat and linked its repressive activity to Tat mutants’ transcriptional strength. Together, these results support a Tat-dependent, proximal mechanism: p400 is recruited to active HIV chromatin independently of Tat, but once Tat is present, it suppresses elongation by binding Tat’s basic domain, disrupting Tat–TAR RNA association, and limiting p-TEFb-mediated RNAPII Ser2 phosphorylation at the HIV LTR.

## Material and methods

### Cell lines and cell culture

HEK293T cells (ATCC) were maintained in high-glucose DMEM (Gibco) supplemented with 10% heat-inactivated FBS (Atlas Biologicals, #FS-0500-AD) and PSG (penicillin 100 U/mL, streptomycin 100 µg/mL, L-glutamine 2 mM; ThermoFisher, #10378016). Jurkat CD4⁺ T cells, J-Lat 10.6 (NIH HIV Reagent Program, ARP-9849), Jurkat A2 (ARP-9854, contributed by Dr. Eric Verdin) [24], and Jurkat-derived HIV-integrated cells were maintained in RPMI 1640 (ThermoFisher, #11875093) supplemented with 10% FBS and PSG. Viral reactivation was induced with TNF-α (Cell Signaling Technology, #8902SC), TSA (Sigma, #T-1952), SAHA (LC Laboratories, #V-8477), PMA (Fisher, #BP6851), or flavopiridol (Selleckchem, #S1230) for the indicated times. Cell counts and viability were determined using a Cellometer Auto T4 (Nexcelom) and trypan blue exclusion.

### Plasmids

shRNA expression vectors pMKO.1-puro (Addgene, #8452) and pLKO-Tet-on (Addgene, #21915) were used for cloning, with gene-specific sequences listed in Supplementary Table S1. EP400-Flag ORF was cloned into pCMV6-Entry (Origene, #RG221066). HA-tagged DMAP1 was cloned into pcDNA3.1(+), and DMAP1 cDNA was also cloned into IRES-mCherry (Addgene, #80139) for Jurkat overexpression. pFL-Tat(86)-Flag and deletion mutants were described previously [50]; additional mutants were generated by site-directed mutagenesis. RNAPII RPB1 plasmids (WT, ΔCTD 1-52, ΔCTD 1-30) were obtained from Addgene (#35175, #35176, #35179). GST-tagged DMAP1 was generated in pGEX-4T3 (GE Healthcare, #27-4583-01). HIV_GKO_ dual-fluorescence vector (Addgene, #112234) was obtained from Addgene. HIV-Crimson was built by inserting E2-Crimson into pNL4-3ΔEnv (HRP-20281, BEI Resources), in-frame with nef. HIV_GKO_-ΔTat and Tat mutants were generated by excising the Tat ORF from HIV_GKO_ with BamHI and SalI, cloning into pCMV-Tag2B, mutating by site-directed mutagenesis, and subcloning back into HIV_GKO_. Mutations were verified by Sanger sequencing.

### HIV NL4-3 virus, HIV_GKO_ pseudovirus, lentivirus, and retrovirus production

HIV particles were produced by transfection of NL4-3 into HEK293T, and the collected virus were titrated by p24 ELISA. HIV-Crimson was packaged by transfecting the pNL4-3ΔEnv-Crimson with pCMV-VSV-G (Addgene, #8454) (5:1) into 293T cells. Vector pMKO-puro (Addgene, #8452) and pLKO-Tet-on (Addgene, #21915) expressing gene-specific shRNA were cotransfected with pCMV-VSV-G and pUMVC (Addgene, #8449) at the ratio of 9:1:8 in HEK293T cells for pseudotyped retrovirus packaging. HIV_GKO_ was packaged in HEK293T cells by transfection of HIV_GKO_ (Addgene, #112234) and pCMV-VSV-G (Addgene, #8454) expression plasmid at a ratio of 5:1. Vector IRES-mCherry expressing DMAP1 was co-transfected with pCMV-VSV-G and CMV-intron at the ratio of 10:1:9 in HEK293T cells for pseudotyped retrovirus package. All the pseudotyped retroviruses were titrated by infection of the Jurkat CD4^+^ T cell for 3 days.

### Generation of virus-infected primary CD4^+^ T cells and EP400 depletion by siRNA

Virus-infected central memory T cells were generated as previously described by Bosque Lab [51]. Briefly, naïve CD4^+^ T cells were isolated from HIV-1-negative blood donors using magnetic isolation (Naive CD4^+^ T Cell Isolation Kit II, Miltenyi Biotec). Naïve CD4^+^ T cells were activated with anti-CD3/CD28 Dynabeads (1:1 ratio of cells to beads) in the presence of anti-human IL-4, anti-human IL-12, and transforming growth factor β1 (TGF-β1) (1 μg/mL, 2 μg/mL, and 10 ng/mL, respectively, from Peprotech). Cells were plated in 96-well round-bottom plates at a density of 0.5 × 10^6^/mL in RPMI medium supplemented with 10% FBS, penicillin/streptomycin, and L-Glutamine (complete RPMI medium) for 3 days. Afterwards, anti-CD3/CD28 beads were removed, and cells were resuspended and kept at a density of 1 × 10^6^/mL in complete RPMI with 30 IU/mL of IL2 (STEMCELL). Cells were infected on day 7 of culture using HIV-Crimson (1000 ng p24 per million cells) by spinoculation at 1740 × g for 2 h at 37°C. T-cell activation was performed by anti-CD3/CD28 Dynabeads (1:1 ratio of cells to beads in a 96-well U-bottom cell culture plate) or PHAP (20 μg/mL) for 3 days. Virus-infected cells were electroporated with gene-specific siRNA or scramble siRNA control on 5 dpi using Gene Pulser Xcell^TM^ Electroporation system (Bio-Rad) according to the manual. Every 10 million cells were resuspended in 200 μL OPTI-MEM and added with 2 μL siRNA (100 μM). Electroporation was performed in a 4 mm Gap Cuvette with the following conditions: Voltage of 500 V, Pulse Width of 2 ms, 1 Pulses, and Unipolar. 27mer, Dicer-substrate RNAs (DsiRNA) were predesigned and ordered from IDT. siScramble: 51-01-19-09; siEP400: GCAUACAAUGGACUUUCUUAUCUUUAGAAAGUCCA; siBRD4-1: ACCGAGAUCAUGAUAGUCUUGCCUGGACUAUCAIGA; siBRD4-2: GGAAAACAACUAUUACUGAGCAUUCCAGUAAUAGUU. Cells were harvested 48-72 hours after electroporation for downstream analyses.

### Viral infection and retrovirus transductions

For HIV NL43 infection, the NL43 virus was treated with DNase (ThermoFisher, #AM2238) at 1 h at 37°C (20 U/mL) to remove the plasmid contamination, and every 5 ng of p24 HIV NL43 particles was used to infect 1 million Jurkat cells in 0.5 mL medium. The medium was replaced at 8 h post-infection (hpi). After 3 washes of the cell with PBS, the cell is placed back in fresh medium. Viral replication level was determined by either analysis of cell-associated mRNA or HIV-1 p24 gag production in the supernatant. HIV_GKO_ pseudovirus was used to infect Jurkat cells at the M.O.I. (multiplicity of infection) of 0.1, and the medium was replaced at 8 hpi. The cell was seeded back with fresh medium after being washed with PBS. HIV replication level was determined by flow cytometry at the indicated time post-infection. For shRNA-mediated RNAi in Jurkat cells or 293T cells, pseudo-typed retrovirus of pMKO-puro or lentivirus of pLKO-Tet-on was used to transduce cells at the M.O.I. of 1, and the medium was replaced at 8 hpi, and the cell was seeded back with fresh medium. Selection was performed by puromycin (ThermoFisher, #A1113803) at 1 µg/ml for 4-6 days. shRNA expression in pLKO-Tet-On-transduced cells was induced with doxycycline (100 ng/mL) for 48 hours. For the DMAP1 overexpression in J-Lat 10.6, a pseudo-typed retrovirus of IRES-mCherry was used to transduce cells at an M.O.I. of 1 by spinoculation (2000 × g, 2 h at 33°C), and the medium was replaced at 8 hpi. The transduced cells (mCherry positive) were isolated by flow cytometry (BD FACSAria Fusion) on day 2 post-transduction and seeded back for further cell culture. HIV expression level was determined by flow cytometry (BD LSR II) at the indicated times.

### Generation of HIVGKO and HIVGKO-ΔTat integrated populations and single cell clones

Viral particles of HIV_GKO_, HIV_GKO_-ΔTat, and mutants were used to infect Jurkat cells at the M.O.I. of 0.1, and the virus-integrated population (mKO2^+^ cells) was sorted with flow cytometry (BD FACSAria Fusion). The single cell suspensions of integrated cells (mKO2^+^) were cloned by limited dilution to obtain the single cell-derived clone. After 2-3 weeks, surviving clones were screened by flow cytometry to determine the integration (mKO2^+^) and expression (mKO2^+^GFP^+^) or silence (mKO2^+^GFP^−^) of HIV. Selected clones were subjected to the HIV integrated site analysis by reverse PCR [52]. The integration site of HIV_GKO_ clones α10 is at Chr1:121264146. For cDNA analysis, cells were treated with PMA (Phorbol myristate acetate, 20 nM for 16 h) to induce viral mRNA expression before cellular mRNA extraction. Viral cDNA was synthesized using Oligo dT, and the Tat coding region was amplified (Vif-F: GGGTCAGGGAGTCTCCATAGAAT, Tat-R: CTTCTTGTGGGTTGGGGTCTGT) and subjected to Sanger sequencing.

### RNA isolation, reverse transcription, and quantitative RT-PCR

Total RNA was extracted from cells using the RNeasy kit (Qiagen, #74106). RNA was DNase-treated using the TURBO DNA-*free*^TM^ kit (Invitrogen, #AM1907). cDNA was synthesized using random hexamer primers and SuperScript III First Strand Synthesis kit (Invitrogen, #18080051). qPCR was performed using SensiFAST^TM^ SYBR® No-ROX Kit (BioLine, #BIO-98020). Samples were run in triplicate. Target gene mRNA expression was normalized to GAPDH mRNA expression, and the relative abundance was calculated (ΔΔCt) (Supplementary Table S2).

### Quantification of HIV integrated events

Genomic DNA of a viral-infected cell was prepared with the DNeasy blood and tissue kit (Qiagen, #69506). Integration events were quantified by Alu-Gag PCR, followed by nested RT- PCR with primers to the gag region as described previously [9].

### Chromatin immunoprecipitation (ChIP)

Because crosslinking and sonication can disrupt the integrity of large chromatin remodeling complexes such as p400 or mask epitopes required for immunoprecipitation, we primarily employed native ChIP (N-ChIP) to assess p400 recruitment. N-ChIP provided stronger and more reproducible signals, consistent with p400’s ability to engage chromatin through histone variant recognition (e.g. H2A.Z, H3.3). To ensure methodological rigor, we also performed cross-linked ChIP (X-ChIP) for RNAPII after p400 depletion, which reproduced the N-ChIP results. Together, this dual approach allowed us to select the technically optimal method while validating key findings across complementary assays.

X-ChIP was performed as previously described [50]. Native N-ChIP was performed as previously described with some modifications [53]. Every 10 millions of cells were washed by 10 mL ice cold PBS and resuspended in 1 mL buffer 1 (15 mM Tris-HCl, pH 7.5, 300 mM Sucrose, 0.1 mM EDTA, 5 mM MgCl_2_, 15 mM NaCl, 60 mM KCl, 0.5 mM DTT, 0.1 mM PMSF, 5 mM Sodium butyrate), and lyzed with 1 mL buffer 2 (15 mM Tris-HCl, pH 7.5, 300 mM Sucrose, 0.4% NP-40, 0.1 mM EDTA, 5 mM MgCl_2_, 15 mM NaCl, 60 mM KCl, 0.5 mM DTT, 0.1 mM PMSF, 5 mM Sodium butyrate) on ice for 7 min, and then neutralized by 8 mL buffer 3 (15 mM Tris-HCl, pH 7.5, 1200 mM Sucrose, 0.1 mM EDTA, 5 mM MgCl_2_, 15 mM NaCl, 60 mM KCl, 0.5 mM DTT, 0.1 mM PMSF, 5 mM Sodium butyrate). The nuclei-containing pellets were collected by centrifuge (2000 × g, 20 min, 4°C) and suspended in 500 µl MNase digestion buffer (50 mM Tris-HCl, pH 7.5, 300 mM Sucrose, 1 mM CaCl_2_, 4 mM MgCl_2_, 0.2 mM PMSF, 5 mM Sodium butyrate). 0.5 units of Micrococcal Nuclease (New England Biolabs, #M0247S) per million nuclei were added, and chromatin was digested at 37°C for 6 min and quenched by adding 20 μl 0.5 M EDTA. After centrifuge (13000 × g, 10 min, 4°C), supernatant (S1) was kept and the pellet (P1) was resuspended in 100 µl dialysis buffer (1 mM Tris-HCl, pH7.5, 0.2 mM EDTA, 0.2 mM PMSF, 5 mM Sodium butyrate) and dialyzed at 4°C overnight in Slide-A-Lyzer™ MINI Dialysis Device (Thermo Scientific^TM^, #88401). The next day, the dialysis products were centrifuged (13000 × g, 10 min, 4°C), and the supernatant (S2) was kept. S1 and S2 were combined and centrifuged (13000 × g, 10 min, 4°C) three times, and the supernatant was kept each time. DNA purification of 20 μl was used to verify the presence of mono-to-penta nucleosomes by running on a 3% agarose gel. A total of 20 μg DNA was used for each IP in antibody incubation buffer (20 mM Tris-HCl, pH7.5, 150 mM NaCl, 5 mM EDTA, 1% Triton-X 100, 0.2 mM PMSF, 20 mM Sodium butyrate) with 2 μg of the indicated antibody or normal mouse IgG (Millipore, #NI03) and rabbit IgG (Fisher Scientific, #02-6102). The equivalent of 4% chromatin was saved as an input control. Antibodies were immunoprecipitated by adding 15 μl Dynabeads™ Protein A (ThermoFisher Scientific, #100001D) per 4 μg antibody. After 4 washes with the antibody incubation buffers, the DNA binds on beads and input DNA were digested with 100 μg/mL RNase A (ThermoFisher, #R1253) in 50 μL elution buffer (20 mM Tris-HCl, pH7.5, 1% SDS, 50 mM NaCl, 10 mM EDTA, 5 mM Sodium butyrate) at 37°C for 15 min, and then 200 μg/mL proteinase K (ThermoFisher, #EO0491) in another 50 μL elution buffers at 55°C for 20 min. The input DNA and eluted DNA for the beads were purified using a PCR purification kit (Qiagen, #28106) and detected by qPCR (Supplementary Table S3). The relative proportions of coimmunoprecipitated DNA fragments were determined based on the threshold cycle (CT) for each qPCR product. The data sets were normalized to input values (percent input = 2^CT Input-CT IP ^× dilution factor). The average value of the IgG background for each primer was subtracted from the raw data.

### Protein purification, *in vitro* binding experiment, coimmunoprecipitation (Co-IP), and western blot (WB)

#### Protein purification

The plasmids pGEX-4T3 encoding GST-fused protein DMAP1 were expressed in *E. coli* BL 21. Recombinant proteins were induced with isopropyl IPTG and purified with Glutathione Agarose beads (ThermoFisher, #16101) according to the protocol. His-tagged Tat proteins were expressed and purified as described previously [50]. After the purification, 10 µg purified protein was loaded for SDS-PAGE and stained with Coomassie blue.

#### In vitro binding experiment

1 µg purified His tagged Tat protein was mixed with 10 µg GST-tagged DMAP1 proteins in binding/wash (25 mM Tris-HCl, pH 7.2, 150 mM NaCl, 1% Triton-X 100, 0.1% SDS) and incubated at 4°C for 1h, then 1 µg Anti-His tag antibody (ThermoFisher, #MA1-21315) was added to the mixture and incubated 1 h at 4°C, the antibody was immobilized by Dynabeads™ Protein G for Immunoprecipitation (ThermoFisher, #10003D). After extensive 5 times of washes with the binding/wash buffers, the elution was subjected to WB analysis.

#### Tat-TAR RNA interaction assay

Biotinylated TAR-RNA (5′-GGGUCUCUCUGGUUAGACCAGAUCUGAGCCUGGGAGCUCUCUGGCUAACUAGGGAACCC-3′) was synthesized by Integrated DNA Technologies (Coralville, Iowa). A slurry (20 μl) of streptavidin magnetic beads (Invitrogen, #88816) was block for 30 min with 32 μg yeast tRNA (Invitrogen, AM7119) and 50 μg BSA (New ENGLAND Biolabs, #B9000S) in Binding buffer (20 mM Tris-HCl pH 7.5, 2.5 mM MgCl_2_, 100 mM NaCl) and then incubated with 5 μg of biotinylated TAR RNA on a rotating platform at 4°C. Whole cell lysates (500 μg protein total) from stable 293 T cells depleted with EP400 or DMAP1 transiently expressing Tat were used. After taking 2% of the mixture as the input, the lysates were added to the beads in 250 μl TAK buffer (50 mM Tris-HCl, pH 8.0, 5 mM MgCl_2_, 5 mM MnCl_2_, 10 μM ZnSO_4_, 1 mM DTT, 100 mM NaCl) and rotated for 2 h at 4°C. Beads were washed in TNN buffer (50 mM Tris-HCl, pH 7.5, 150 mM NaCl, 1% NP-40) five times and eluted in 40 μl 2 × SDS-loading buffer to resolve on SDS-PAGE. Immunoblot was probed with indicated antibodies as previously described [54].

#### IP

Cells were lysed with IP buffer (50 mM Tris-HCl pH7.5, 1 mM EDTA, 150 mM KCl, 1% Triton-X 100) with protease inhibitor cocktail (Roche, #04693124001) for 20 min on ice, followed by 20 seconds of sonication (Qsonica sonicators, 70% Ampl), and the lysate were centrifuged at 12 000 rpm for 10 min at 4°C. The protein concentration in the supernatant was quantified by Bradford assay (Bio-Rad, #5000006). DNase (Invitrogen, #AM1907) was added to the lysate before incubating with antibody (20 U/mL) if indicated. One mg of cell lysate was incubated with 4 µg of the indicated antibody overnight at 4°C, then an additional 1 h of incubation with 15 µL Dynabeads^TM^ Protein G (Invitrogen, #01009761) at 4°C. After five washes with IP buffer, beads were resuspended in SDS loading buffer (Bio-Rad, #1610747), and Co-IP proteins were separated on an SDS-PAGE gel and identified by WB.

#### Western blot (WB)

For the detection of the expression of EP400, DMAP1, and BRD4 by WB, the nuclei of stable cells was isolated. Cells and nucleus were added with RIPA buffer supplemented with complete EDTA-free protease inhibitor cocktail (Roche, #04293132001), and 500 mM NaCl was added to prevent the contamination of DNA. Lysate centrifuged at 12 000 rpm for 10 min at 4°C. Protein concentration determined by Bradford assay. Total protein extracts were separated by SDS-PAGE and transferred onto polyvinylidene difluoride membranes. Membranes were probed with indicated antibodies (Supplementary Table 4) followed by horseradish peroxidase-conjugated secondary antibody (Supplementary Table 4). The membranes are exposed with ECL^TM^ prime Luminol Enhancer solutions (Cytiva), recorded and analysed by Imagine Lab software (Bio-Rad Laboratories). The GAPDH was used as the loading control for whole cell lysate, and H3 or HDAC1 was used as the loading control for the nucleus.

#### ELISA

Quantification of HIV p24 capsid production was performed using the antigen capture assay kit from Advanced BioScience Laboratories, Inc. (ABL, #5447), according to the manufacturer’s protocol.

#### Flow cytometry analysis and Statistical analysis

Flow cytometry data were analyzed using FlowJo v10, and graphs were generated with GraphPad Prism v10.0. Data are presented as mean ± SEM. Statistical significance between groups was assessed using paired *t*-tests, one-way ANOVA, or two-way ANOVA, as appropriate. *P* < 0.05, *P* < 0.01, *P* < 0.001, and *P* < 0.0001; values of *P* < 0.05 were considered statistically significant.

## Results

### Screening of ATPase subunits of human chromatin remodeling complexes in latency models reveals EP400 as a repressor of HIV transcription

**Figure 1.**
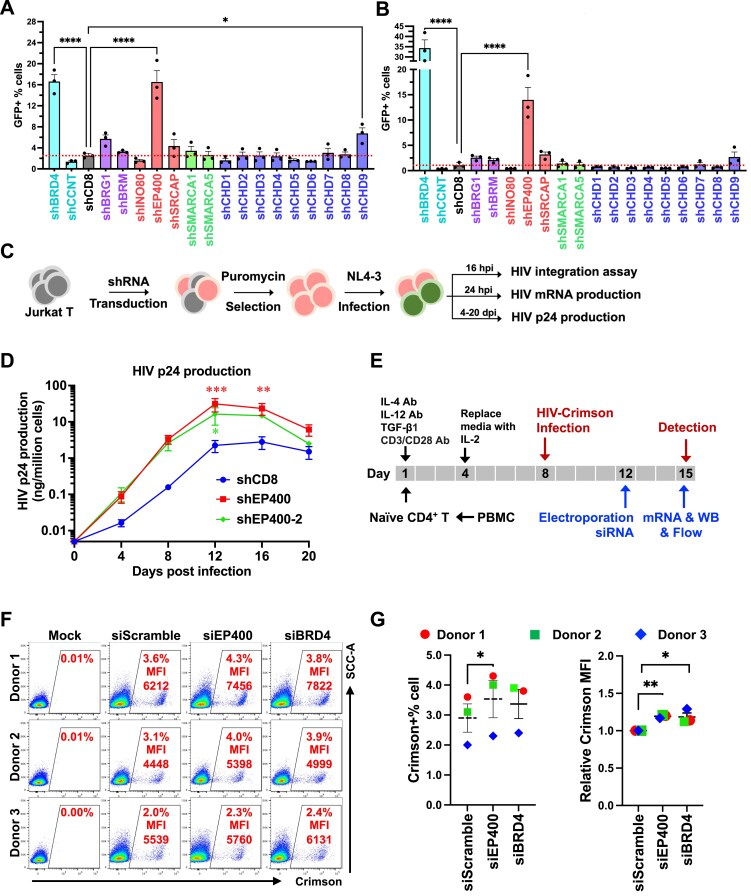
Screening ATPase subunits of human chromatin remodeling complexes in latency models identifies EP400 as a repressor of HIV transcription. **(A-B)** HIV gene expression, measured as the percentage of GFP⁺ cells, in J-Lat 10.6 **(A)** and Jurkat A2 **(B)** cells stably depleted of ATPase subunits from human CRCs. Cells were transduced with gene-specific shRNAs and selected with puromycin (1 µg/mL) for 6 days. **(C)** Workflow for stable Jurkat T-cell selection, HIV infection, and analysis of viral replication at DNA, mRNA, or protein levels. EP400 or CD8 shRNAs were introduced as in (A-B), and cells were infected with HIV NL4-3 (5 ng p24 Gag per 10^6^ cells) and washed at 8 hpi. **(D)** HIV p24 production in the stable Jurkat cell lines described in **(C). (E)** Workflow for HIV-Crimson infection of primary CD4⁺ T cells with siRNA-mediated depletion. **(F-G)** Flow cytometry plots showing crimson fluorescence upon EP400 or BRD4 depletion **(F)**, and a summary of Crimson+% and MFI across three donors **(G)**. Data in **(A, B, D, G)** represent mean ± SEM from three independent experiments. Statistical significance was assessed by one-way ANOVA **(A-B)**, two-way ANOVA **(D)**, or paired t-test **(G**).

To examine the roles of the 16 human CRCs in HIV transcription (Supplementary Fig. S1A), we employed two widely used HIV latency models, J-Lat 10.6 cells and Jurkat A2, both derived from the T-cell leukemia Jurkat cell line [24]. J-Lat 10.6 cells contain a full‐length, replication‐defective HIV provirus in which the envelope gene is replaced by GFP, whereas Jurkat A2 cells carry a mini‐HIV genome with the LTR driving Tat‐IRES‐eGFP expression (Supplementary Fig. S1B and C). In both models, latency reversal agents (LRAs) induce a robust, quantifiable fluorescence signal (Supplementary Fig. S1B and C). We generated stable knockdowns of each of the 16 ATPase subunits in both models using gene-specific shRNAs followed by puromycin selection (Supplementary Fig. S1D and S1F). HIV transcription was measured by GFP fluorescence (Fig. 1A and B) and Gag mRNA levels (Supplementary Fig. S1E). CCNT1, a core component of the p-TEFb complex essential for HIV transcription [4], was included as a positive control, whereas BRD4, which suppresses HIV transcription by restricting Tat’s access to p-TEFb and by cooperating with SWI/SNF to repress HIV transcription [55−57], served as a negative control. Depletion of BRG1, EP400, SRCAP, and CHD9 resulted in a significant induction of HIV transcription (Fig. 1A and B and Supplementary Fig. S1E), consistent with previously reported repressive functions of SWI/SNF (BRG1/BRM) and CHD9 in HIV latency [11, 23]. Importantly, p400 and SRCAP, both members of the INO80 family remodelers, were identified as a negative regulator of HIV gene expression. In contrast, depletion of INO80A, CHD1, CHD5, or CHD6 markedly reduced HIV transcription, phenocopying CCNT1 depletion and implicating these factors as positive regulators (Fig. 1A and B and Supplementary Fig. S1E).

EP400 was prioritized for follow-up studies because its knockdown produced the strongest reactivation, boosting GFP⁺ cells from ∼2% to ∼16% and increasing gag mRNA ∼25-fold. The bulk mRNA increase reflects mean transcript levels across all cells, whereas flow cytometry measures the fraction exiting latency; together, these data indicate strong per-cell transcriptional upregulation within a reactivating subpopulation, consistent with the known heterogeneity of latent HIV [58]. We next assessed EP400 function during acute HIV infection in Jurkat T cells using the NL4‐3 isolate (Fig. 1C). While EP400 knockdown did not alter HIV integration levels (Supplementary Fig. S2A and B), it significantly increased viral mRNA production (Supplementary Fig. S2C) and capsid p24 production (Fig. 1D). We extended the analyses to primary central memory CD4⁺ T cells (Tcm) infected with a single‐cycle VSV‐G-pseudotyped HIV‐crimson virus (Supplementary Fig. S2D). Infection reached 16% infection at 3 days post-infection (dpi) (Supplementary Fig. S2E and F), but Crimson fluorescence declined thereafter and did not rebound with T‐cell activation (Supplementary Fig. S2F and G), indicating productive rather than latent infection; the loss of signal is most consistent with cytopathic effects in cells with high viral protein expression. EP400 knockdown in infected Tcm from three donors, confirmed at both mRNA and protein levels (Supplementary Fig. S2H and I), significantly increased HIV-crimson fluorescence (Fig. 1F and G) and total HIV transcripts (Supplementary Fig. S2J) in each donor, indicating that EP400 functions as a negative regulator of HIV transcription.

### p400 negatively regulates HIV latency establishment and reactivation

**Figure 2.**
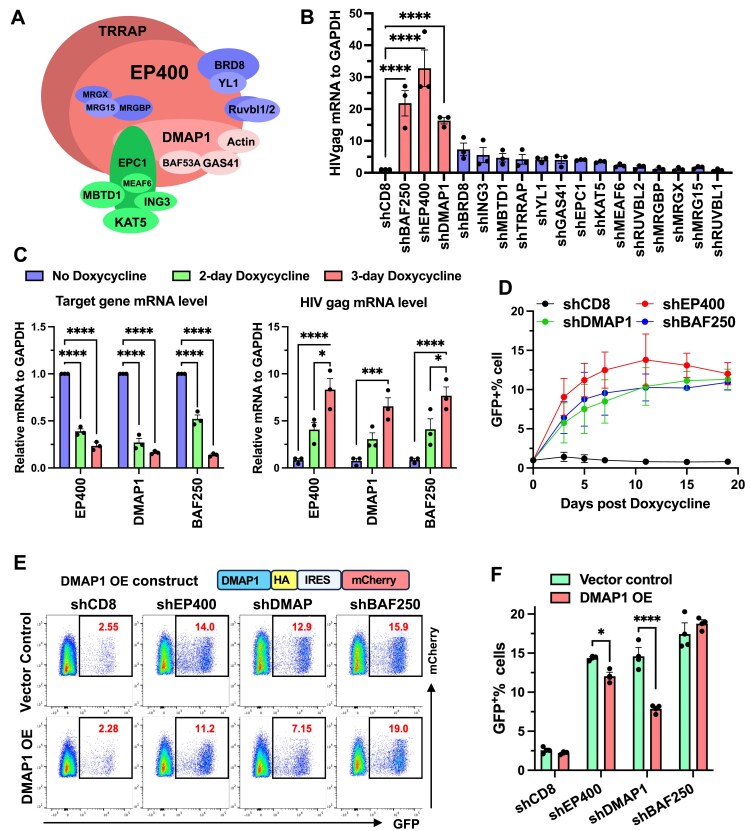
p400 negatively regulates HIV latency establishment and reactivation. **(A)** Schematic representation of the human p400 complex. **(B)** Relative HIV mRNA levels in stable J-Lat 10.6 cells following depletion of the indicated p400 subunits. Cells were transduced with retroviral vectors expressing gene-specific shRNAs and selected with puromycin (1 µg/mL) for 6 days prior to analysis. **(C)** HIV expression in J-Lat 10.6 cells after doxycycline-induced shRNA expression, measured by mRNA (C) or GFP^+^% **(D). (E** and **F)** HIV expression (GFP⁺%) in J-Lat 10.6 cells stably depleted of EP400, DMAP1, or BAF250, with or without DMAP1 overexpression. Overexpression was achieved by transduction with VLPs carrying DMAP1 cDNA or vector control, and GFP⁺% was measured 10 days post-transduction. Data in (B, C, D, F) represent mean ± SEM from three independent experiments. Statistical significance was assessed by one-way ANOVA (B) or two-way ANOVA (C, F).

We next depleted additional subunits of the p400 complex in J‐Lat 10.6 cells using shRNAs (Supplementary Fig. S3A) and measured effects on HIV transcription (Fig. 2A). BAF250, a BAF complex subunit that promotes nuc‐1 deposition downstream of the HIV TSS and suppresses viral activation [11], served as a repressive control. Knockdown of most p400 components induced a ∼4–30‐fold increase in HIV reactivation, except for MEAF6, MRG15, MRGX, MRGBP, RUVBL1, and RUVBL2 (Fig. 2B). This pattern supports a modular p400 architecture in which a subset of core subunits directly enforces transcriptional HIV repression, whereas shared factors such as RUVBL1/2, present in multiple remodeling complexes, likely play broader structural roles with little HIV-specific impact. Depletion of the core subunits EP400 or DMAP1 produced the strongest reactivation and was therefore used to model p400 complex loss in subsequent assays. Notably, EP400 or DMAP1 knockdown also potentiated LRA-induced viral gene expression across a broad range of concentrations (Supplementary Fig. S3B and C).

To minimize confounding from prolonged RNAi, we used a doxycycline-inducible shRNA system for acute depletion of EP400 or DMAP1. Cells were transduced with retroviral vectors, selected with puromycin for 4 days, and shRNA expression was induced with doxycycline. Within 48 h of induction, EP400, DMAP1, and BAF250 transcripts were markedly reduced (Fig. 2C), coincident with increased HIV gag mRNA and GFP that rose further by day 3 and continued through day 15 (Fig. 2C and D). These short-window (48–72 h) perturbations mitigate long-term adaptation and reproduce the Tat-dependent, RNAPII Ser2 phosphorylation-focused phenotype; sustained increases at later time points, and potential indirect contributions, are addressed in the companion study [59].

To test the specificity of the HIV-repressive phenotype caused by p400 loss, we performed a genetic rescue. Because EP400 cDNA exceeds our retroviral packaging capacity, we engineered a shRNA-resistant DMAP1 cDNA in a bicistronic IRES-mCherry vector and transduced J-Lat 10.6 cells. DMAP1 overexpression (OE) alone did not alter GFP expression (Supplementary Fig. S3D and E), indicating that DMAP1 by itself cannot recapitulate the structure or function of the intact p400 complex. In cells with stable EP400 or DMAP1 knockdown, however, DMAP1 OE partially restored repression, reducing reactivation by approximately 50% relative to DMAP1 knockdown, whereas no rescue was observed after BAF250 depletion (Fig. 2E and F). These data support a specific role for the EP400-containing p400 complex in repressing HIV transcription and suggest that full activity requires the intact complex.

### The p400 subunit DMAP1 interacts with Tat, while EP400 engages the RNAPII CTD

**Figure 3.**
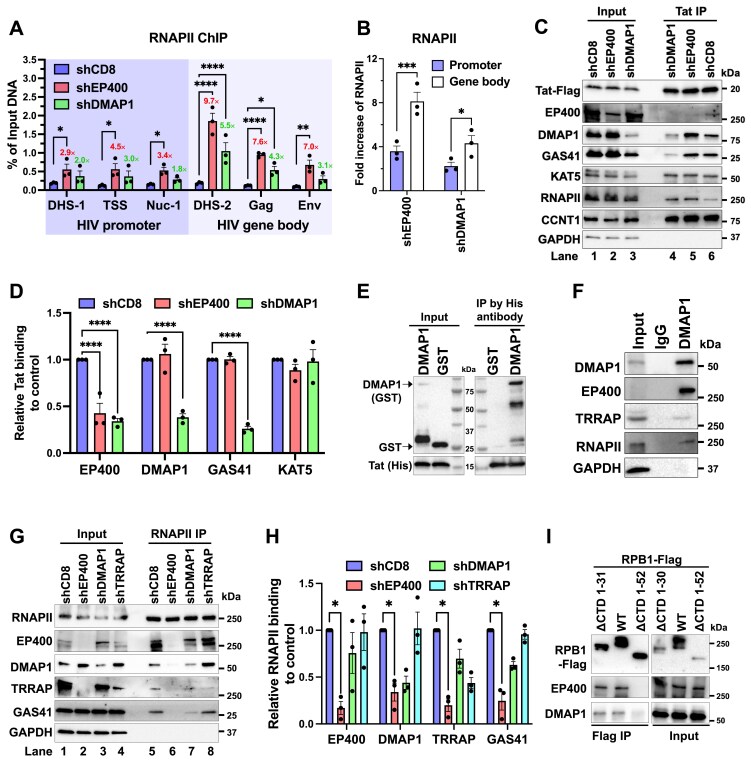
p400 subunit DMAP1 interacts with Tat, while EP400 associates with the RNAPII CTD. **(A** and **B)** N-ChIP for RNAPII in J-Lat 10.6 cells following EP400 or DMAP1 depletion, shown as % input DNA with fold-change indicated. **(C** and **D)** Representative co-immunoprecipitation (Co-IP) of endogenous p400 and RNAPII in stable 293T cells depleted of EP400 or DMAP1 (C) and quantification from three independent experiments (D). The sample loading order differs between the Input and IP panels, as lanes were adjusted to minimize edge effects in SDS-PAGE and to aid visualization. Relative Tat binding was quantified from copurified Tat band intensities normalized to Tat IP band from the same sample. **(E)**  *In vitro* binding assay between recombinant purified His-Tat and GST-DMAP1. **(F)** Co-IP of endogenous p400 and RNAPII in Jurkat T cells using anti-DMAP1 antibody. **(G** and **H)** Co-IP of RNAPII and p400 in 293T cells depleted of EP400, DMAP1, or TRRAP (G) and quantification from three experiments (H). **(I)** Co-IP of overexpressed RPB1 with endogenous p400 in 293T cells. Data in (A, B, D, H) represent mean ± SEM from three independent experiments. Statistical analysis was performed using two-way ANOVA with multiple comparisons against the control.

To define how p400 constrains HIV transcription, we mapped RNAPII occupancy by N-ChIP-qPCR at the HIV locus in J-Lat 10.6 cells. Using J-Lat10.6 specific primers (Supplementary Fig. S4A), EP400 or DMAP1 knockdown significantly increased RNAPII occupancy across the HIV locus (Fig. 3A), with no change at an active control gene (ENO1), or a silent locus (CA6) (Supplementary Fig. S4B). The increase was substantially greater over the HIV gene body than at the promoter (EP400: ∼8.1-fold versus ∼3.6-fold; DMAP1: ∼4.3-fold versus ∼2.2-fold) (Fig. 3B), consistent with p400 restricting promoter escape/early elongation. These findings prompted further investigation into the mechanistic basis of p400-mediated repression, particularly its interaction with Tat.

Multiple p400 subunits have been reported as Tat interactors by mass spectrometry (MS), including EP400, DMAP1, TRRAP, BAF53A, RUVBL1, RUVBL2, GAS41, and KAT5 [60]. In HEK293T cells, Tat co-purified with RNAPII, KAT5, DMAP1, EP400, and GAS41 (Supplementary Fig. S4C). Stable knockdown of DMAP1 significantly reduced Tat co-purification of EP400 and GAS41 (as well as DMAP1 itself), whereas EP400 knockdown did not affect Tat-DMAP1 or Tat-GAS41 associations (Fig. 3C and D), indicating that p400 engages Tat through DMAP1. Consistently, depletion of other p400 subunits identified by MS did not disrupt Tat-DAMP1 binding (Supplementary Fig. S4D and E). Neither DMAP1 nor EP400 altered the Tat-KAT5 association, and KAT5 depletion did not disrupt Tat-DMAP1 binding, suggesting that KAT5 interacts with Tat independently. Finally, a direct DMAP1-Tat interaction was confirmed by GST pull-down using purified GST-DMAP1 and His-Tat protein (Fig. 3E; Supplementary Fig. S4F and G), demonstrating that p400 contacts Tat through DMAP1.

Co‐IP in Jurkat cells showed that immunoprecipitation of p400 via DMAP1 co‐purified RNAPII (Fig. 3F). The same p400-RNAPII association was observed in HEK293T cells when DMAP1 or EP400 was immunoprecipitated (Supplementary Fig. S4H and I), indicating that the interaction is conserved across cell types. DNase treatment did not diminish recovery of RNAPII or p400 components and yielded minimal H3 co‐purification, confirming the association is not DNA-mediated (Supplementary Fig. S4H and I). Reciprocal RNAPII immunoprecipitation co‐purified multiple p400 subunits, including EP400, DMAP1, TRRAP, and GAS41 (Fig. 3G, lanes 1 and 5). EP400 depletion markedly reduced RNAPII co‐purification with these subunits, whereas DMAP1 or TRRAP depletion had no effect (Fig. 3G and H), identifying EP400 as the principal bridge between p400 and RNAPII. Moreover, the EP400–RNAPII association required the RNAPII CTD, as RPB1 constructs lacking the CTD (ΔCTD1-52; Supplementary Fig. S4J) failed to co‐purify EP400 or DMAP1 (Fig. 3I). Together, these findings support a model in which p400 binds Tat through DMAP1 and connects to RNAPII via EP400 recognition of the CTD.

### DMAP-1-mediated suppression of HIV transcription depends on concurrent interactions between EP400 and Tat

**Figure 4.**
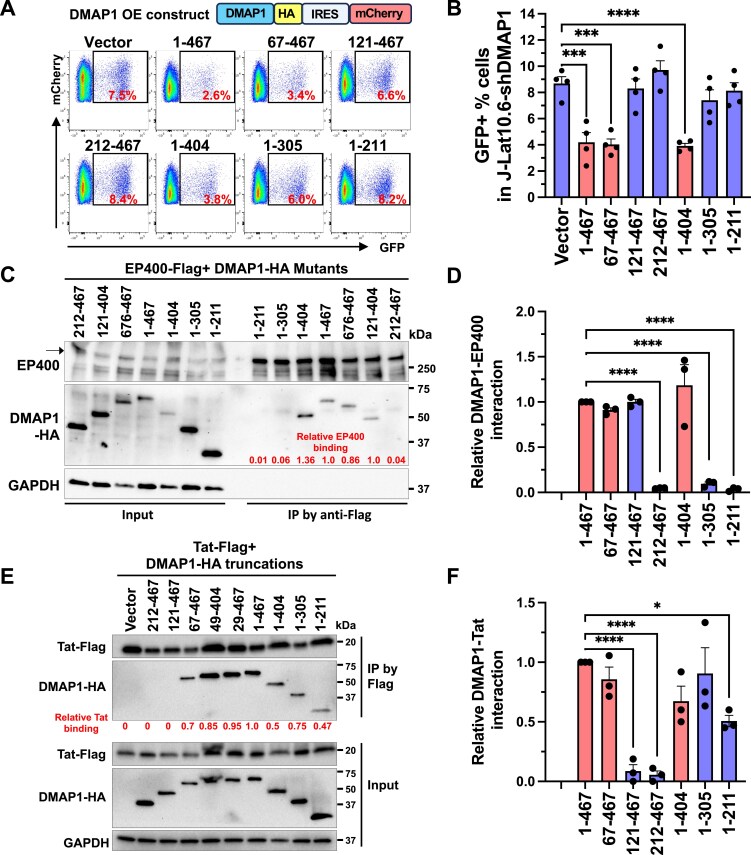
HIV suppression requires simultaneous DMAP1, EP400, and Tat interactions. **(A-B)** GFP^+^% in J-Lat 10.6 cells depleted of DMAP1 and complemented with DMAP1 truncations, shown by representative flow plots (A) and quantification from four experiments (B). The *y*-axis represents the expression of DMAP1, and the *x*-axis represents the HIV gene expression level. **(C-D)** Co-IP of Flag-tagged EP400 with DMAP1 truncations in 293T cells (C) and quantification from three experiments (D). The sample loading order differs between the Input and IP panels, as lanes were adjusted to minimize edge effects in SDS-PAGE and to aid visualization. **(E** and **F)** Co-IP of Flag-tagged Tat with DMAP1 truncations in 293T cells (E) and quantification from three experiments (F). Data in (B) are mean ± STE from four experiments; data in (D, F) are mean ± SEM from three experiments. Statistical significance was assessed by one-way ANOVA (B, D, F).

In J‐Lat 10.6 cells, the increase in HIV transcription caused by DMAP1 depletion was rescued by OE of full‐length DMAP1 (WT; Fig. 2E and F). To map the repressive domains, we performed rescue assays with DMAP1 truncations in cells with stable DMAP1 knockdown and quantified HIV reactivation by flow cytometry. Full‐length DMAP1 and constructs spanning residues 67-467 and 1-404 effectively suppressed HIV transcription, whereas smaller fragments were inactive (Fig. 4A and B). Co‐IP analyses revealed that the active constructs (WT [1-467], 67-467, 1-404) retained binding to both EP400 and Tat (Fig. 4C–F). In contrast, fragments that lacked Tat interaction (121-467, 212-467) or EP400 interaction (1-305, 1-211) failed to suppress HIV transcription (Fig. 4). Thus, effective repression of HIV transcriptional elongation by DMAP1 requires simultaneous engagement of both EP400 and Tat.

### p400 specifically suppresses Tat-dependent HIV replication and transcription

**Figure 5.**
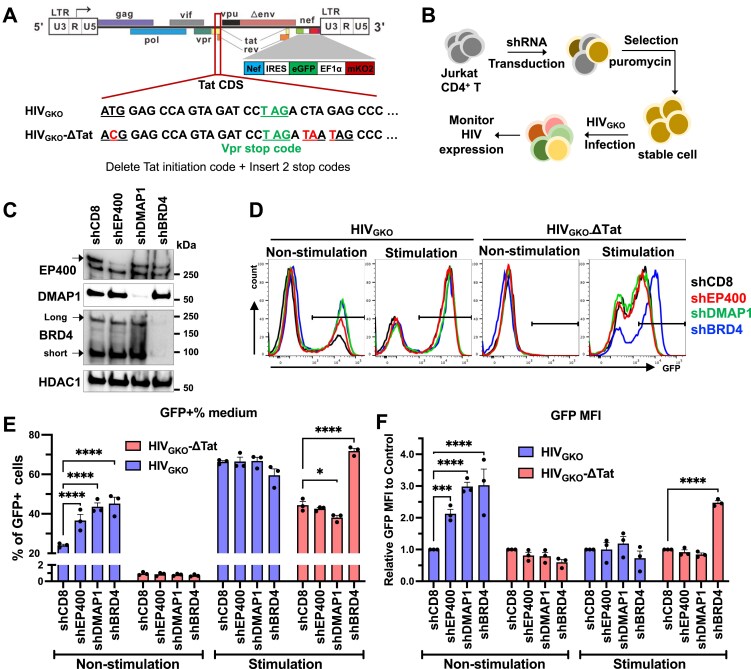
p400 specifically suppresses HIV Tat-dependent viral replication. **(A)** HIV_GKO_ vector and its Tat-deficient variant (HIV_GKO_-ΔTat). **(B)** Workflow for stable Jurkat T-cell selection, HIV_GKO_ infection, and analysis of viral replication. **(C)** Western blot of EP400, DMAP1, and BRD4 in stable Jurkat cells depleted by shRNA. **(D-F)** Representative flow cytometry plots of GFP expression (D) with quantification of GFP^+^% (E) and GFP MFI (F). Cells were infected at MOI = 0.1 (day 1), reactivated with PMA + TSA (day 3), and analyzed on day 4. HIV_GKO_-ΔTat infected cells were stimulated with 10 nM PMA + 2 µM TSA, while HIV_GKO_ infected cells received 10% of that dose to avoid overstimulation. Data in (E-F) represent mean ± SEM from three experiments. Statistical significance was assessed by two-way ANOVA.

To test whether p400 restrains Tat-dependent elongation, we engineered a Tat-deficient variant of the dual‐reporter HIV vector HIV_GKO_ [49], termed HIV_GKO_‐ΔTat, by mutating the Tat start codon (ATG→ACG), introducing a synonymous Vpr change, and adding two stop codons downstream of the Vpr coding region (Fig. 5A). Jurkat T cells stably depleted of EP400, DMAP1, or the control factor BRD4 were infected with VSV‐G-pseudotyped HIV_GKO_ or HIV_GKO_-ΔTat (Fig. 5B and C). Viral transcription was induced with LRAs at 3dpi, and HIV expression quantified on 4dpi after excluding cells without integrated provirus (mKO2-) (Supplementary Fig. S5A). In HIV_GKO_-infected cells, EP400 and DMAP1 depletion increased the proportion of actively transcribing cells (GFP⁺%) and GFP mean fluorescence intensity (MFI) without affecting cell viability or integration level (Fig. 5E and F and Supplementary Fig. S5B and C). In contrast, with HIV_GKO_-ΔTat, EP400 or DMAP1 depletion failed to enhance viral transcription, with or without LRA treatment (Fig. 5E and F). BRD4 depletion increased transcription in both HIV_GKO_ and HIV_GKO_‐ΔTat infections (Fig. 5E and F), consistent with its role in antagonizing Tat-p‐TEFb engagement and recruiting repressive SWI/SNF complexes [55, 56, 57]. Notably, in HIV_GKO_-infected cells, LRAs did not further elevate GFP⁺ frequency or GFP MFI upon p400 or BRD4 depletion, consistent with near-maximal activation during acute infection and potential cytotoxic limits.

**Figure 6.**
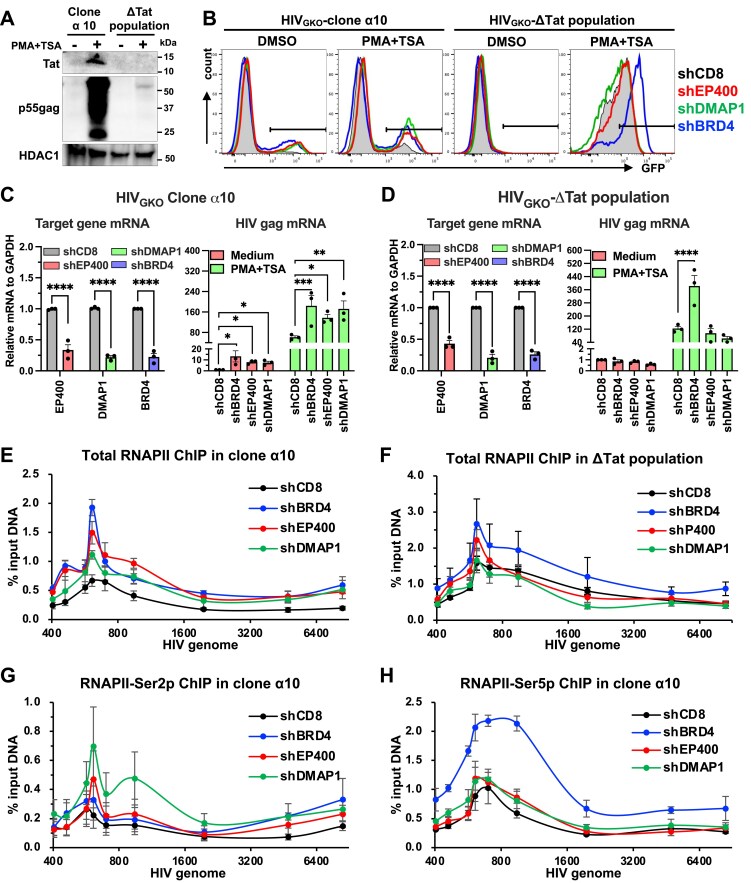
p400 suppresses HIV Tat-dependent viral activation. **(A)** Western blot of Tat and Gag p55 expression in HIV_GKO_ clone α10 and HIV_GKO_-ΔTat populations. Cells were treated with 10 nM PMA + 2 µM TSA to reactivate HIV gene expression. **(B–D)** GFP expression in HIV_GKO_ clone α10 and HIV_GKO_-ΔTat populations depleted of EP400, DMAP1, or BRD4, shown by flow plots (B), mRNA analysis of host genes (C), and HIV transcripts (D). Cells were transduced with retroviral vectors expressing gene-specific shRNAs, followed by puromycin selection (1 µg/mL) for 4 days before analysis. HIV_GKO_-ΔTat cells were stimulated with 10 nM PMA + 2 µM TSA, while HIV_GKO_ clone α10 cells received 10% of that dose to avoid overstimulation. **(E–H)** X-ChIP-qPCR analysis of total RNAPII in HIV_GKO_ clone α10 cells (E) and HIV_GKO_-ΔTat cells (F), and of RNAPII-Ser2P (G) and RNAPII-Ser5P (H) in HIV_GKO_ clone α10 cells following depletion of EP400, DMAP1, or BRD4.

Flow cytometry-based single‐cell sorting of HIV_GKO_‐infected populations yielded a Tat-competent clonal line (HIV_GKO_ clone α10), and an HIV_GKO_‐ΔTat population (HIV_GKO_‐ΔTat). Both displayed low basal HIV expression that was inducible with LRAs (Supplementary Fig. S6A). Tat competence in clone α10 and Tat deficiency in HIV_GKO_‐ΔTat were confirmed by Tat cDNA sequencing and Tat‐specific immunoblotting (Supplementary Fig. S6B, Fig. 6A). As expected, loss of Tat in the HIV_GKO_‐ΔTat population led to minimal viral gene expression even after PMA + TSA treatment, as indicated by low GFP signal (Supplementary Fig. S6A) and minimal p55 Gag accumulation (Fig. 6A), underscoring Tat’s essential role in driving robust HIV transcription. Stable depletion of EP400, DMAP1, or BRD4 was performed in both cell types, and viral gene expression was quantified by GFP reporter and gag mRNA levels (Fig. 6B–D). In clone α10, EP400 or DMAP1 depletion significantly increased gag mRNA with or without LRA treatment, whereas in the HIV_GKO_‐ΔTat population, the same depletions caused a slight, non‐significant reduction in gag mRNA, consistent with GFP readouts (Fig. 6B–D). As expected, BRD4 depletion significantly enhanced viral gene expression in both contexts.

We performed X-ChIP for RNAPII to validate the specific role of the p400 complex in HIV transcription, using PMA + TSA-treated samples from Fig. 6B–D using HIV_GKO_-specific primers (Supplementary Fig. S6C). Consistent with GFP reporter and HIV gag mRNA, depletion of EP400 or DMAP1 increased total RNAPII recruitment at the HIV locus in HIV_GKO_ clone α10, but not in the HIV_GKO_‐ΔTat population (Fig. 6E-F). X-ChIP-qPCR for RNAPII-ser2p and RNAPII-Ser5p further showed that EP400 and DMAP1 depletion selectively enhanced RNAPII-Ser2P occupancy at the HIV locus (Fig. 6F and G). As a control, BRD4 depletion increased total RNAII recruitment at the HIV locus, with elevations in both RNAPII-Ser5p and RNAPII-Ser2p occupancy. As expected, EP400 and DMAP1 depletion did not change RNAPII occupancy in HIV_GKO_‐ΔTat population, whereas BRD4 depletion still increased RNAPII occupancy (Fig. 6H).

Collectively, these data support that the p400 complex, acting with DMAP1, specifically restrains Tat-driven RNAPII Ser2P-associated elongation and thereby suppresses HIV-1 gene expression, with negligible impact in the absence of Tat.

### Recruitment of p400 to transcriptionally active HIV loci is Tat-independent

As previously observed by ChIP-seq [59], p400 is enriched at transcriptionally active HIV loci, seemingly at odds with its negative role in Tat‐dependent transcription. To probe this, we reactivated HIV transcription with TNF‐α and subsequently inhibited transcription using the RNAPII CTD kinase inhibitor flavopiridol for 1 h. N-ChIP revealed that RNAPII occupancy at both the HIV promoter and gene body increased upon TNF‐α stimulation and decreased following flavopiridol treatment (Supplementary Fig. 7A). EP400 and DMAP1 exhibited similar dynamics, rising with activation and falling upon inhibition (Supplementary Fig. S7B and C). ChIP specificity was validated in J‐Lat 10.6 cells stably depleted of EP400 and DMAP1, which showed reduced signal at both the HIV genome and the ENO1 TSS (Supplementary Fig. S7D and E). Moreover, EP400 depletion lowered DMAP1 occupancy at HIV and ENO1, and vice versa, indicating reciprocal dependence consistent with their cooperative assembly within the p400 complex.

**Figure 7.**
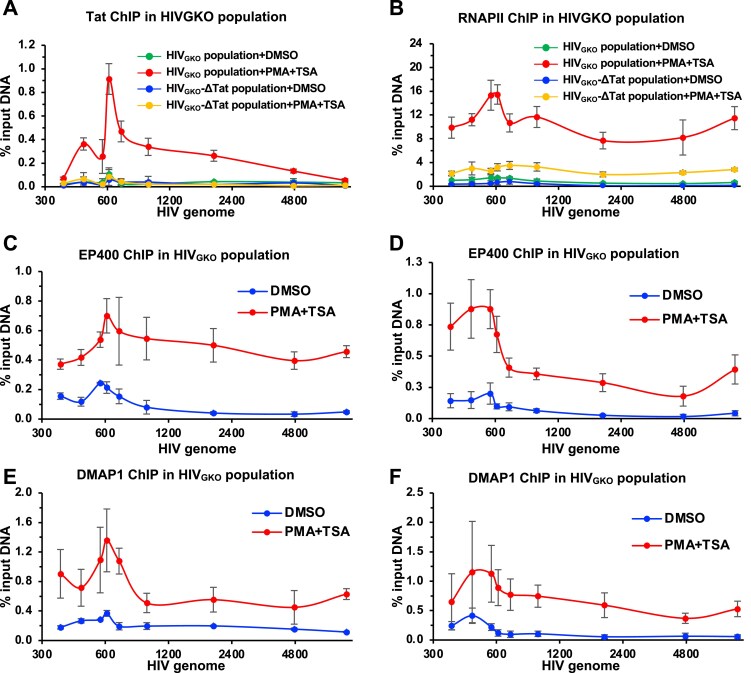
Recruitment of p400 to transcriptionally active HIV loci is Tat-independent. **(A-B)** N-ChIP for RNAPII (A) and Tat (B) in Jurkat cells with HIV_GKO_ or HIV_GKO_-ΔTat. **(C-D)** N-ChIP for EP400 in HIV_GKO_ (C) or HIV_GKO_-ΔTat (D) populations. **(E-F)** N-ChIP for DMAP1 in HIV_GKO_ (E) or HIV_GKO_-ΔTat (F) populations. For all experiments, cells were treated with 10 nM PMA + 2 µM TSA to reactivate HIV gene expression. Data represent mean ± SEM from three independent experiments.

To test whether Tat mediates p400 recruitment to active HIV loci, we performed N-ChIP for RNAPII, p400, and Tat in HIV_GKO_ and HIV_GKO_-ΔTat populations (Supplementary Fig. S7F), with Tat expression verified by Western blotting (Supplementary Fig. S7G). Tat ChIP-qPCR showed robust Tat occupancy at the HIV locus in HIV_GKO_ but not in HIV_GKO_-ΔTat (Fig. 7A). RNAPII recruitment increased markedly upon PMA + TSA activation in HIV_GKO_ and, to a lesser extent, in HIV_GKO_‐ΔTat population (Fig. 7B). In contrast, EP400 and DMAP1 recruitment was comparable between HIV_GKO_ and HIV_GKO_‐ΔTat populations and increased strongly after PMA + TSA in both. Thus, despite physical interaction between p400 and Tat, p400 is recruited to transcriptionally active HIV independently of Tat.

Collectively, these findings indicate that p400/DMAP1 recruitment to HIV chromatin is driven by transcriptional activity and is Tat-independent, whereas repression of elongation is Tat-dependent, reconciling their enrichment at active proviruses with selective restraint of Tat-driven transcription.

### p400 engages Tat’s basic domain and disrupts Tat-TAR binding, thereby restricting transcriptional elongation

**Figure 8.**
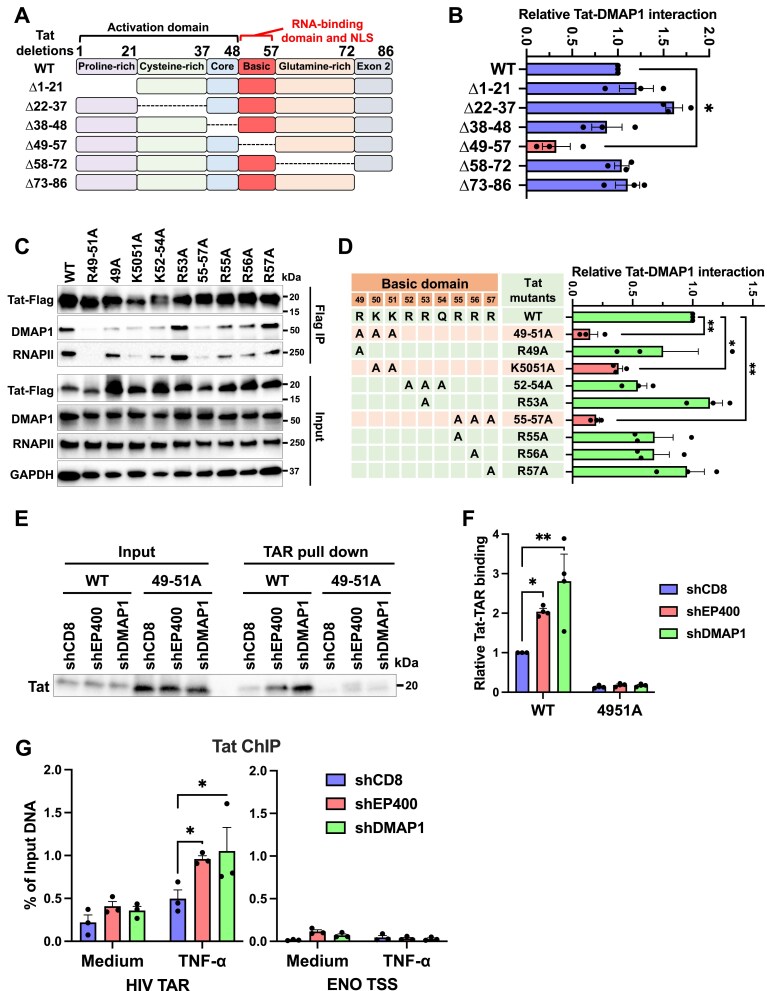
p400 interacts with Tat’s basic domain and interferes with Tat–TAR interaction. **(A)** Schematic representation of Tat deletion variants. **(B)** Tat-DMAP1 interaction by Co-IP in 293T cells, quantified by DMAP1 band intensity normalized to Tat IP band. **(C** and **D)** Co-IP of Tat mutants with DMAP1 and RNAPII (C) and quantification from three experiments (D). Relative Tat-DMAP1 interaction was calculated as the DMAP1 band intensity normalized to Tat input. **(E** and **F)** Tat–TAR interaction in 293T cells depleted of EP400 or DMAP1, shown by immunoblot (E) and quantification from three experiments (F). Interaction was quantified as the TAR-copurified Tat band intensity normalized to Tat input. **(G)** Native ChIP for Tat in HIVGKO clone α10 cells following EP400 or DMAP1 depletion, using HIV TAR-specific primers and ENO TSS primers as controls. Data in (B, D, F, G) represent mean ± SEM from three experiments. Statistical significance was assessed by one-way ANOVA (B, D) or two-way ANOVA (F, G).

To define the Tat interface for DMAP1 binding, we generated Tat deletion mutants (Fig. 8A) and performed co‐IP assays with overexpressed DMAP1. Deletions of residues 1-21 or 22-37 reduced Tat protein levels, consistent with decreased stability (Supplementary Fig. S8A). Importantly, deletion of the basic domain (Δ49-57) markedly diminished Tat-DMAP1 binding (Fig. 8B and Supplementary Fig. S8A), identifying this region as essential for the interaction. By comparison, the Tat core mediated KAT5 binding, as its deletion selectively reduced Tat–KAT5 association (Supplementary Fig. S8B), indicating distinct interfaces for DMAP1 and KAT5. Point mutagenesis within Tat’s basic region (Fig. 8D) showed that single amino acid substitutions (R49A, R53A, R55A, R56A, and R57A) did not impair DMAP1 binding, whereas double/triple substitutions (49-51A, K50-51A, and 55-57A) significantly weakened it (Fig. 8C and D), demonstrating cooperative contributions of multiple basic residues.

Because Tat’s basic domain mediates HIV TAR RNA binding, we asked whether p400 competes with TAR for Tat. Biotin‐labelled TAR RNA pull‐downs using HEK293T lysates expressing WT or mutant Tat confirmed loss of TAR binding by the BRM mutant (all Arg→Ala) [61] and reduced TAR interaction for F38A, T40A, 38–40A, 49–51A, 52–54A, and 55–57A (Supplementary Fig. S8C and D), consistent with prior reports [62−64].

Depleting EP400 or DMAP1 (Supplementary Fig. S8E) significantly increased WT Tat-TAR binding, but not the 49–51A mutant, which is defective for DMAP1 interaction (Fig. 8E and F; Supplementary Fig. S8G). Tat ChIP in Jurkat clone α10 further showed that EP400 or DMAP1 depletion enhanced Tat occupancy at the HIV locus without affecting the ENO1 TSS control (Fig. 8G). Consistently, basic domain mutations reduced Tat-RNPII co-IP (Fig. 8C), underscoring the requirement of this region for Tat-RNAPII interaction. EP400 or DMAP1 depletion increased Tat-RNAPII association by more than 2-fold, while Tat-CCNT1 interaction remained unchanged (Supplementary Fig. S8F and G). Collectively, these data support a model in which p400, via DMAP1, sequesters Tat’s basic domain, limiting TAR-Tat engagement and downstream RNAPII Ser2 phosphorylation.

**Figure 9.**
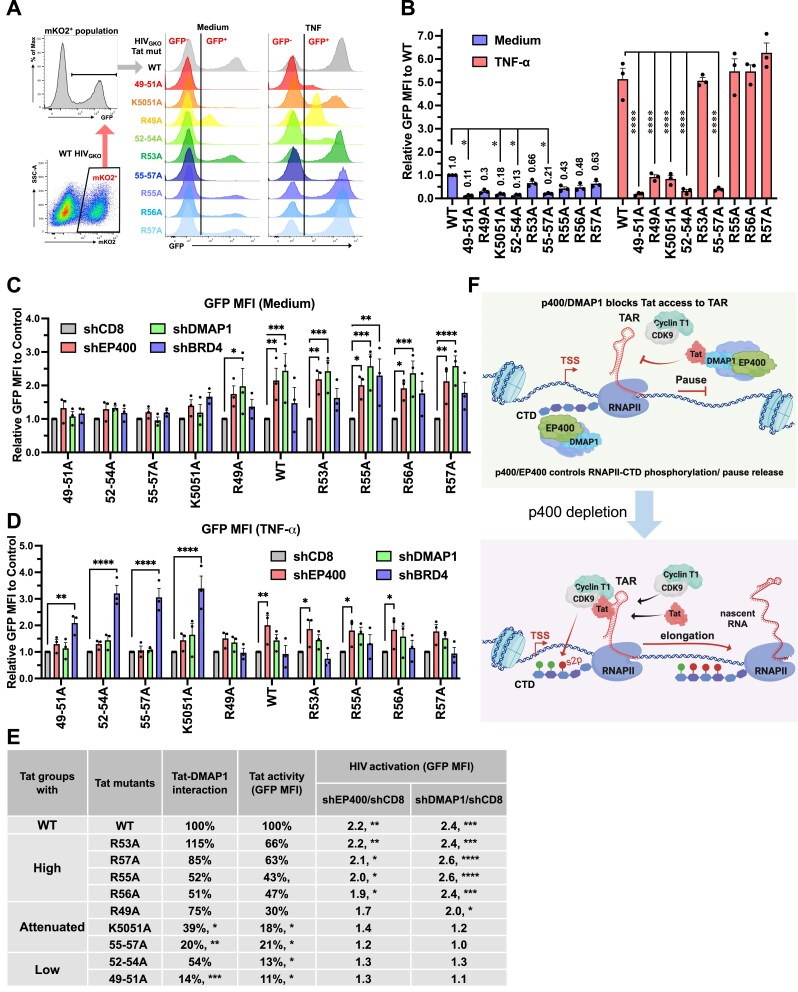
p400 restricts Tat-dependent HIV gene expression. **(A)** Flow cytometry plot of GFP expression in Jurkat T cells infected with HIV_GKO_ Tat mutant viruses. Viral integration was gated on mKO2^+^ cells. **(B)** Quantification of GFP MFI in HIV_GKO_-integrated populations in (A). **(C-D)** GFP MFI of Tat-mutated HIV_GKO_ virus in stable Jurkat T cells depleted of EP400, DMAP1, or BRD4. Cells were infected at MOI = 0.3 (day 1), treated with TNF-α (day 3), and analyzed on day 4. **(E)** Properties of alanine substitutions in the Tat basic domain: DMAP1 binding (from Fig. 8D), transactivation activity (from B), and HIV activation after EP400 or DMAP1 depletion (from C). **(F)** Model illustrating the role of the p400 complex in HIV transcriptional regulation. Data in (B–D) represent mean ± SEM from three experiments. Statistical significance was assessed by two-way ANOVA.

To test functional consequences in infection, we engineered HIV_GKO_ vectors encoding Tat basic-domain mutants and assessed Tat activity by GFP readouts in infected (mKO2⁺) cells (Fig. 9A). Based on the GFP MFI, R53A, R55A, R56A, and R57A retained substantial activity (>47% of WT Tat), whereas R49A, K50-51A, and 55-57A were attenuated (18–30% of WT), and 52–54A and 49–51A exhibited minimal activity (<15% of WT) (Fig. 9B; Supplementary Fig. S9A). In Jurkat cells, stable depletion of EP400, DMAP1, or BRD4 significantly increased basal transcription of WT and high-activity mutants (R53A, R55A, R56A, R57A) (1.9–2.6‐fold), whereas minimal-activity mutants (49–51A, 52–54A) were largely unresponsive to EP400/DMAP1 loss (1.0–1.3‐fold) (Fig. 9C and E). Upon TNF‐α stimulation, minimal-activity mutants were reactivated by BRD4 depletion (∼3‐fold by GFP MFI) but remained unresponsive to EP400 or DMAP1 depletion (Fig. 9D). Across mutants, higher Tat activity correlated with stronger repression by EP400/DMAP1.

Notably, R49A (30% of WT activity; 75% of WT DMAP1 binding) showed significant reactivation upon p400 depletion (1.7–2.0‐fold), whereas K50/51A (18% of WT activity, 39% of WT DMAP1 binding) was less responsive (1.2–1.4‐fold) (Fig. 9E), underscoring the requirement for Tat-DMAP1 engagement. A modest increase (<30%) in minimal-activity mutants upon EP400 depletion (Fig. 9C and E) suggests a Tat‐independent component, likely via p400‐regulated host genes [59]. Collectively, these data are consistent with DMAP1 engaging Tat’s basic domain to limit Tat-TAR binding and RNAPII-Ser2 phosphorylation, restricting transcriptional elongation (Fig. 9F).

## Discussion

Chromatin remodeling by SWI/SNF complexes has been extensively studied in the context of HIV transcription [10, 11, 15, 65, 66], but the roles of other chromatin remodeling complexes (CRCs) remain less defined. To address this gap, we conducted an shRNA screen targeting the 16 human SNF2-family ATPases in J-Lat 10.6 and Jurkat A2 latency models (Fig. 1 and Supplementary Fig. S1). All three INO80/SWR family complexes (p400, SRCAP, and INO80) modulate HIV transcription, with p400 and SRCAP acting as repressors and INO80 as an activator. Modest effects from INO80, CCNT1, and several CHD family members (e.g. CHD1, CHD5, CHD6) likely reflect the limited dynamic range of these latency systems, underscoring the need for models with higher basal transcription to better resolve positive regulators. Focusing on the p400 complex, we identified EP400 and DMAP1 as critical subunits mediating Tat-dependent HIV repression. Our data are consistent with a Tat-proximal mechanism in which DMAP1 engages Tat basic domain to limit Tat-TAR binding and RNAPII Ser 2 phosphorylation.

We distinguish recruitment from function and the conditions under which each occurs. Recruitment of p400/DMAP1 to transcriptionally active HIV chromatin is Tat-independent and most consistent with recognition of RNAPII and ongoing transcription, whereas repression is Tat-dependent and manifests during reactivation once Tat accumulates. We do not claim that p400 enforces repression in the complete absence of Tat; rather, p400 restrains elongation when Tat is present.

Although structural information for human p400 is still emerging, insights from yeast NuA4 complex, which shares 12 of its 13 subunits with the human counterpart, are informative [35]. NuA4 comprises the Tra1/TRRAP subunit; the HAT module (Eas1/KAT5, Yng2/ING3, Eaf6/MEAF6); the TINTIN module (Eaf3/MRG15, Eaf5/no human homolog, Eaf7/MRGBP); and the core module (Eaf1/EP400, Eaf2/DMAP1, Yaf9/GAS41, Arp4/BAF53A, Act1/Actin) that governs assembly [35]. Within the core module, Eaf1/EP400 serves as the structural scaffold [32−34], a role recently confirmed by cryo-EM analysis of the human p400 complex [30, 31]. Consistent with these roles, EP400 depletion strongly derepressed latent HIV, likely destabilizing complex integrity and the p400-Tat interface, whereas DMAP1 depletion primarily impaired the DMAP1-Tat engagement, yielding a stricter Tat-dependent phenotype (Fig. 2A and B). A recent CRISPR latency screen likewise flagged EP400 and DMAP1 as top NuA4 subunits whose loss reactivates HIV across multiple J-Lat models [67]. While KAT5-mediated H4 acetylation can recruit BRD4 to restrict Tat-p-TEFb [68], our data support a distinct pathway: p400 limits HIV elongation independently of BRD4, underscoring multiple conserved routes to proviral repression.

Canonical HAT and histone-variant activities of p400’s HAT module (KAT5/ING3/MEAF6) [33, 69], did not phenocopy EP400 or DMAP1 loss (Fig. 2A and B), and Ac-H4, H3.3, and H2A.Z occupancy at the HIV locus were unchanged by EP400/DMAP1 depletion [59], indicating that classical HAT or variant deposition is not the primary mechanism here. While KAT5 has been reported to bind Tat’s N-terminus, we found this interaction to be independent of DMAP1 and distinct from Tat-DMAP1 binding (Fig. 3C and D, Supplementary Fig. S8B). KAT5 can enhance Tat-dependent transcription [70, 71], yet Tat can paradoxically suppress KAT5’s HAT activity and promote its degradation [72−74]. Notably, the DMAP1 region required for Tat binding (aa 67–121) corresponds to the yeast Eaf2 segment that interacts with actin and Arp4 [34], suggesting that Tat may destabilize p400 structural integrity and NuA4 HAT function by concurrently targeting DMAP1 and KAT5. These observations underscore the compositional and contextual plasticity of EP400-containing assemblies.

Our findings, together with our recently published work [59], indicate that p400 is recruited to transcriptionally active HIV loci independent of Tat (Fig. 7), likely via RNAPII-CTD association (Fig. 3I), and its occupancy is reduced upon transcriptional inhibition by flavopiridol (Supplementary Fig. S7A–C). DMAP1 depletion reduced RNAPII co-purification with EP400 (Fig. 3G-H) and decreased EP400 occupancy at HIV and ENO1 (Supplementary Fig. S7D), suggesting partial dependence on this interaction. We propose a two-phase model: in the absence of Tat, p400 can modestly support HIV transcription (consistent with slight reductions in HIV mRNA upon EP400/DMAP1 depletion in HIV_GKO_‐ΔTat infected cells) (Fig. 6B–D), a role shared at host genes via EP400-RNAPII interaction [59]. Once Tat accumulates, however, p400’s dominant role shifts to repressing Tat transactivation (Fig. 9F). Because HIV transcription occurs in discrete bursts, with alternating phases of activity and silence [75], and Tat amplifies burst amplitude and frequency by recruiting P-TEFb, p400 functions as a post-initiation brake that modulates burst size by attenuating Tat activity. During active transcription, p400 binds Tat via DMAP1, competes with TAR RNA, and limits RNAPII processivity. Upon p400 depletion, this restraint is removed, strengthening Tat-TAR engagement and positive feedback.

To mitigate long-term p400 loss adaptation, acute perturbations reproduced the core phenotype: doxycycline-inducible shRNA in Jurkat cells (48–72 h) and siRNA in primary CD4^+^ T cells (48–72 h) yielded a significant increase in viral infection or transcription activation (Figs 1F–G and 2C). Functionally, reactivation following p400 or DMAP1 depletion was strictly Tat-dependent (lost in Tat-KO contexts and in Tat basic-domain mutants that weaken DMAP1 binding) (Figs 5–6, 8–9). DMAP1 truncation mutants that retained both EP400 and Tat interactions rescued repression after knockdown, whereas truncations that lost either interaction did not (Fig. 4). Consistent with a selective effect on transcription elongation, p400 loss increased RNAPII Ser2 phosphorylation without comparable elevation of phosphorylation over Ser5 (Fig. 6). These lines of evidence support a Tat-dependent, proximal mechanism in which p400 limits elongation once Tat is present.

In primary CD4⁺ T cells, EP400 depletion produced consistent but modest increases in HIV transcription, likely reflecting the pre-activated nature of the model and use of a replication-incompetent reporter virus. Even so, a ∼40–50% increase is biologically meaningful given the tight HIV transcriptional control, and the effect was reproducible across independent donors. Effects were most evident under basal conditions and diminished after LRA stimulation (Fig. 9C and D and Supplementary Fig. S3C), consistent with abundant Tat exceeding p400’s repressive capacity and LRAs engaging parallel pathways (e.g. NF-κB) that bypass Tat dependence. Cytotoxicity from high viral protein expression may further deplete strongly reactivated cells, constraining measurable responses. No *in vitro* system fully captures the complexity of HIV latency *in vivo* [76]. Jurkat models and pre-activated primary T cells provide mechanistic insights but lack the full diversity of integration sites, cell states, and immune pressures present in PLWH. Thus, our findings should be interpreted within these constraints, and validation in reservoir-harboring memory CD4⁺ T cells from ART-suppressed individuals will be essential to confirm the physiological relevance of the p400-Tat axis.

These mechanistic insights have important translational implications. By defining a pathway in which DMAP1 engages the Tat basic domain to restrict transcriptional elongation, we identify a regulatory axis usable in both “shock-and-kill” and “block-and-lock” strategies [77−79]. Enhancing p400’s repressive capacity or stabilizing DMAP1-Tat binding may promote durable silencing, whereas transient disruption could sensitize refractory proviruses to reactivation. Notably, the effects of EP400/DMAP1 depletion parallel those of the Tat inhibitor didehydro-cortistatin A (dCA), which selectively blocks Tat-dependent elongation and promotes durable silencing [9, 80]. While p400’s pleiotropy may limit direct druggability, these findings provide a mechanistic foundation for developing Tat modulators or chromatin-based interventions that exploit this pathway.

## Supplementary Material

gkaf1323_Supplemental_Files

## Data Availability

No new data were generated or analyzed in support of this research.
