# Supplementary material for: The human chromatin remodeling complex p400 restricts HIV-1 transcription in a Tat-dependent manner
Source: Nucleic Acids Res. 2025 Dec 18;53(22):gkaf1323. doi: 10.1093/nar/gkaf1323 (PMC12714564; doi:10.1093/nar/gkaf1323)
Supplement: gkaf1323_Supplemental_Files [file gkaf1323_supplemental_files.zip › supplementary text.docx]

**Supplementary Figure Legends:**

**Figure S1. Screening of ATPase subunits of human chromatin remodeling complexes that regulate HIV transcription and reactivation from latency.**

**(A)** Overview of the 16 human CRCs screened. **(B-C)** Basal and PMA-induced HIV gene expression (GFP^+^%) in J-Lat 10.6 and Jurkat A2 latency models, showing robust induction with PMA treatment. **(D)** Efficient depletion of the indicated ATPase subunits in J-Lat 10.6 cells, confirming knockdown. **(E)** HIV mRNA levels in J-Lat 10.6 cells following ATPase subunit depletion, revealing differential effects of specific CRCs on viral transcription. **(F)** Validation of ATPase subunit knockdown in Jurkat A2 cells.
Data in **(D-F)** are mean ± SEM from three independent experiments. Statistical significance was assessed by one-way ANOVA in **(E)** and two-way ANOVA in **(D, F)**.

**Figure S2. EP400 functions as a negative regulator of HIV transcription.**

**(A-C)** Stable depletion of EP400 in Jurkat T cells confirmed by qPCR for EP400 mRNA **(A)**, Alu-PCR/qPCR for HIV integration at 16 hpi **(B)**, and RT-qPCR for viral mRNA at 24 hpi **(C, as in Fig. 1C)**. **(D)** Schematic of the HIV-Crimson reporter virus. **(E)** Workflow for generating HIV-infected central memory T cells (Tcm). **(F-G)** Flow cytometry monitoring HIV expression (Crimson^+^%) in Tcm **(F)**, with summary across three donors **(G)**. Cells were left untreated or stimulated with anti-CD3/CD28 dynabeads or PHA-P on day 6 post-infection. **(H-I)** EP400 and BRD4 expression in siRNA-transfected Tcm assessed by qPCR **(H)** and Western blot **(I)**. The arrow marks the expected band; * indicates non-specific.
Data in **(A-C, G-H)** are mean ± SEM from three independent experiments. Statistical significance was assessed by one-way ANOVA (**A-B**) and two-way ANOVA (**C, H**).

**Figure S3. p400 suppresses HIV transcription and limits viral reactivation.**

**(A)** Relative mRNA levels of the indicated genes in J-Lat 10.6 cells depleted using gene-specific shRNAs, selected with puromycin (1 µg/mL, 6 days). **(B)** Western blot analysis of EP400, DMAP1, and BAF250 in stably depleted J-Lat 10.6 cells. **(C)** Reactivation of latent HIV in J-Lat 10.6 cells depleted of EP400, DMAP1, or BAF250 after 16 h treatment with the indicated LRAs. Viral expression was measured by flow cytometry (GFP). **(D)** Western blot analysis of DMAP1 overexpression. **(E)** Representative flow cytometry plots of viral expression (GFP) upon DMAP1 overexpression, indicated by mCherry co-expression.
Data in **(A, C)** are mean ± SEM from three independent experiments. Statistical significance was assessed by two-way ANOVA.

**Figure S4. Interactions of p400 subunits with Tat and RNAPII.**

**(A)** HIV-specific primers used for ChIP-qPCR in J-Lat10.6. **(B)** RNAPII recruitment to cellular promoters ENO1-TSS and CA6-TSS, analyzed by ChIP-qPCR. **(C)** Representative IP showing co-purification of p400 subunits and RNAPII with Flag-tagged Tat in 293T cells. **(D)** Relative mRNA levels of the indicated genes in 293T cells depleted of EP400, RUVBL1, RUVBL2, KAT5, or GAS41. **(E)** Representative IP of Flag-Tat in 293T knockdown cells from **(D)**. **(F)** Purified GST and GST-DMAP1 detected by GST and DMAP1 antibodies. **(G)** Purified His-tagged Tat analyzed by Coomassie staining. **(H-I)** Co-IP of endogenous p400 and RNAPII in 293T cells using EP400 antibody **(H)** or DMAP1 antibody **(I)**, with or without DNase treatment. **(J)** Schematic of RNAPII RPB1 constructs: WT, NTD+CTD31-52, and NTD-only. Data in **(B, D)** are mean ± SEM from three independent experiments. Statistical significance was assessed by two-way ANOVA.

**Figure S5. Flow cytometry analysis of HIV_GKO_ replication in Jurkat T cells.**

**(A)** Gating strategy for HIV_GKO_-infected Jurkat T cells. **(B)** Cell viability assessed by live/dead dye. **(C)** Viral integration measured by % mKO2^+^ cells at 3 dpi.

**Figure S6. Viral activation level and Tat cDNA sequencing of HIV_GKO_ and HIVGKO-ΔTat integrated cells.**

**(A)** HIV gene expression in HIV_GKO_ clone α10 and HIV_GKO_-ΔTat populations with or without LRA treatment. Cells were stimulated with 10 nM PMA and 2 µM TSA to induce reactivation. **(B)** Sanger sequencing of Tat cDNA from HIV_GKO_ and HIV_GKO_-ΔTat cells after stimulation with 10 nM PMA and 2 µM TSA. **C.** HIV-specific primers used for ChIP-qPCR in J-Lat10.6. The name corresponds to the midpoint between the two oligos on the HIV genome.

**Figure S7. p400 co-occupies with RNAPII at the active HIV promoter and gene body.**

**(A-C)** Native ChIP for RNAPII **(A)**, EP400 **(B)**, and DMAP1 **(C)** in J-Lat 10.6 cells. Latent HIV was reactivated with TNF-α (10 ng/mL, 16 h) followed by treatment with or without flavopiridol (100 nM, 1h). **(D-E)** Native ChIP for DMAP1 **(D)** and EP400 **(E)** in J-Lat 10.6 cells depleted of EP400 or DMAP1. **(F-G)** HIV gene expression and protein analysis in HIV_GKO_ and HIV_GKO_-ΔTat populations. Cells were stimulated with 10 nM PMA and 2 µM TSA. Viral gene expression was measured **(F)**, and Tat and Gag p55 proteins were assessed by Western blot **(G)**. Data represent mean ± SEM from three independent experiments. Statistical significance was assessed by two-way ANOVA.

**Figure S8. p400 interacts with Tat at the basic domain and disrupts Tat-TAR binding.**

**(A-B)** Co-IP of Flag-tagged Tat deletion mutants with DMAP1 **(A)** or KAT5 **(B)** in 293T cells. **(C-D)** Tat-TAR interaction assay. **(C)** Schematic of Tat pull-down with biotinylated TAR RNA. **(D)** Western blot of Tat variants bound to TAR RNA in 293T cells. **(E)** Western blot showing depletion of EP400 or DMAP1 in 293T cells. **(F-G)** Tat interaction with RNAPII and CCNT1. **(F)** Representative Co-IP of Flag-Tat with RNAPII and CCNT1 in 293T cells. **(G)** Quantification of three experiments showing Tat-RNAPII and Tat CCNT1 interactions normalized to Tat input. Data represent mean ± SEM from three independent experiments. Statistical significance was assessed by two-way ANOVA.

**Figure S9. p400 restricts Tat-dependent viral gene expression.**

**(A)** Relative viral gene expression quantified by GFP^+^% of HIV_GKO_ populations infected with HIV_GKO_ Tat mutant viruses. **(B)** Relative mRNA levels of EP400, DMAP1, and BRD4 in stable Jurkat T cells. **(C)** Representative flow cytometry plots of HIV gene expression in Jurkat T cells stably depleted of EP400, DMAP1, or BRD4 and infected with HIV_GKO_ Tat mutant virus. Cells were infected at MOI = 0.3 on day 1, treated with or without TNF-α (10 ng/mL, 24 h) on day 3, and analyzed by flow cytometry on day 4. Viral integration was gated on mKO2^+^ cells, and viral protein expression was assessed by GFP^+^% and GFP MFI. Data in **(A-B)** are mean ± SEM from three independent experiments. Statistical significance was assessed by two-way ANOVA.

**Supplementary Tables:**

**Table S1. shRNA target sequence.**

**Table S2. Oligos for mRNA level analysis.**

**Table S3. Oligos for ChIP-qPCR analysis.**

**Table S4. Antibodies used in this study.**

**Table S1. shRNA target sequence.**

| shRNA | Target sequence | Cloning Vector |
| --- | --- | --- |
| shCD8 | GGCATCTACTTCTGCATGATC | pMKO.1-puro, pLKO-Tet-on |
| shCCNT1 | TGCATCGATTCTACATGATTC | pMKO.1-puro |
| shBAF250 | CCGCAGGAGCTATCTCAAGAT | pMKO.1-puro, pLKO-Tet-on |
| shBRD4 | CCTGGAGATGACATAGTCTTA | pMKO.1-puro |
| shBRG1 | GAGGAGGAGGAGGAGAAGATG | pMKO.1-puro |
| shBRM | AATGGGCCTCAAAGATTCAGA | pMKO.1-puro |
| shINO80A | AACTTGGTCTCCATTTCATAT | pMKO.1-puro |
| shEP400 | GAGCGCCTGGATCAGATTTAT | pMKO.1-purom pLKO-Tet-on |
| shEP400-2 | CAGGAGCTGTTTGAAGTTTAT | pMKO.1-puro |
| shSRCAP | AAGGCAAATCAGAAGAGAATG | pMKO.1-puro |
| shSMARCA1 | AACTGAGACTCGATTCAATTG | pMKO.1-puro |
| shSMARCA5 | AATGCTCAATTGTTATGTCAT | pMKO.1-puro |
| shCHD1 | AACGAGGCAATCGAGTTCTTA | pMKO.1-puro |
| shCHD2 | AACTTCTATCACAGTTTAAGG | pMKO.1-puro |
| shCHD3 | AAGGGCGTAGACAGTCAAAGA | pMKO.1-puro |
| shCHD4 | AAGTACATCCTCACTCGAAAT | pMKO.1-puro |
| shCHD5 | CGCAACTACCAAAGAAAGAAC | pMKO.1-puro |
| shCHD6 | GACCAGGTAACCAAGGATATT | pMKO.1-puro |
| shCHD7 | AGGGTGCCTGTTGTCAATAAA | pMKO.1-puro |
| shCHD8 | GTCAGCCCCTAAGATTGTTAT | pMKO.1-puro |
| shCHD9 | GAGGGCCTGAAACTCATGAAT | pMKO.1-puro |
| shDMAP1 | GGGCGACGCTGTTATGTAAAT | pMKO.1-puro, pLKO-Tet-on |
| shTRRAP | ATGCTCCAGTTACTTTCAAAT | pMKO.1-puro |
| shGAS41 | TAGGAGCCTATAAGCATGAAA | pMKO.1-puro |
| shKAT5 | TCCTCCAGGCAATGAGATTTA | pMKO.1-puro |
| shEPC1 | ATCCGACCGAAACGGAAATAT | pMKO.1-puro |
| shING3 | GAGAGGCGATCTTTGGAATTA | pMKO.1-puro |
| shMBTD1 | TTGCTGGATGGAGAGGATTAT | pMKO.1-puro |
| shMEAF6 | TGCTGGTATTCACCTGATTAA | pMKO.1-puro |
| shMRG15 | GGCGGCGAATCACTTATAAAT | pMKO.1-puro |
| shMRGX | TCGCAGGGAAATGTTGATAAT | pMKO.1-puro |
| shMRGBP | CAGAGAATTTGTAGCGGTTAT | pMKO.1-puro |
| shBRD8 | GAGCCTAAGGAAGAGGATCAA | pMKO.1-puro |
| shYL1 | CGGGACCCTGTTACAGACATA | pMKO.1-puro |
| shRUVBL1 | TGGCGTCATAGTAGAATTAAT | pMKO.1-puro |
| shRUVBL2 | GACACTGTGACTCTGTATAAA | pMKO.1-puro |

**Table S2. Oligos for mRNA level analysis**

| mRNA Primer | Forward sequence / Reverse sequence |
| --- | --- |
| HIV total mRNA | GGTTAGACCAGATCTGAGCCTGG / CAACAGACGGGCACACACTACT |
| HIV gag mRNA | GCAATGAGCCAAGTAACAAATCCA / CCTTTTTCCTAGGGGCCCTGC |
| GAPDH | CAACAGCCTCAAGATCATCAGCA/AGGGATGACCTTGCCCACAGCCTTGG |
| BAF250A | GAACCTGAGCCTTCAGTCAAGAC/GGTATGAGGAGAGAAAGGAGACTGA |
| BRD4 | TGGATGCCGTCAAGCTGAAC/GTTTCTTCTGTGGGTAGCTCATT |
| CCNT1 | ACAACAAACGGTGGTATTTCACT/CCTGCTGGCGATAAGAAAGTT |
| BRG1 | CCCTCAGGACAACATGCACCA/CTGGACTAGAGGCATGCTCAGA |
| BRM | GGTCCACAGACTTCCCACAGGA/ CAGACATAGGGCTGGAGACGTG |
| INO80A | GAGCAGGCAGTTCTGGAATGT/ GCCTCGCTGGATTCATCACTT |
| EP400 | GAAGCACAGTAGAGACGGACCT/ GTGCGGTCGGCATTAGTTGCT |
| SRCAP | GCCAAGGATGTCAGGCAGTTCT/ CTGGAGGTTAATGGCTGGTTGA |
| SMARCA1-F | CCGCCACTATCGTGGTCATAGA/ GCGCTTTAGCAGCAAGTTTGAGT |
| SMARCA5-F | CTCAACTCAGCAGGCAAGATGGA/ CCATCCAACCTGCAGTACTCAT |
| CHD1 | GGATCCTGACCTCAGTCTAACACA/GGCAGAGGAGAAGAATCACTCTT |
| CHD2 | GGAACGGATAGCTGATGTGAAGA/ CCCACTCTCAGACCCGCTACT |
| CHD3 | GAAACGAAGAAGGAAGCACCGA/ CTCATGAACTGGCTGAAGGCT |
| CHD4 | GGACCCTAAAATCCCTAAGAGCAA/ CCGCTTGGATTTGCTCTTCTTCT |
| CHD5 | CGCCGAGGAGATGGAGAATGA/ CACTGCCTTCACTCTCCGACTT |
| CHD6 | GTGTCCTTGCAGGGTGTCTGA/ CTCTTCTTGATCAGTGCTGCAGT |
| CHD7 | GGGTGCCTGTTGTCAATAAACGA/ CGATCTCACTTTTGGGCCTTCT |
| CHD8 | GCAGGCTCAGATAATGGGACCA/ GCGGTTTTCCTCCATTCCTGT |
| CHD9 | CTCCTTTACCTGGTGAACAGCCT/ TGTGCTCTTTGTGCTTGTCTCAA |
| TRRAP | GTGGTTGACCAGACCACTTTGAT/ CTGTGCTGGTTTCTCCTGAAGA |
| DMAP1 | CTATGCCTTGCTCTACTCTGACAAG/ GTAGTCCTTGCCCTCCTCCGCT |
| GAS41 | GAAACAGGATGGGGTGAATTCGA/CATCATTGCTGTTGGGTCTTGAAA |
| EPC1 | GCCAAAGCAGCTCATTCACATAC/ GTTTGGCTTCCTGCAGACTGAC |
| KAT5 | AACAAACGTCTGGATGAATGGG/ AGGAAGTCCGTTCTTAGTGGG |
| MBTD | CTGTGAGATGTGTGGGATGGTT/ CCAGCTGAAACCTTCCATGGA |
| ING3 | GCTTCACGGAAATGCGCGAGAT/ CCAGCTTTCTCAAGTGTCGATCTA |
| MEAF6 | GGGATCGGTATCTGACCAACCA/ CTGATTGTGGAAGTCTGGAGAAGT |
| YL1 | CAGTGATGGAGAAGCAGAAGAGC/GTCAGAGCCGTCATCTTGTAGTTC |
| BRD8 | GTTAGCAGAGCAATCAAGCCCTT/ CAGTCTGCTGTCCATGTGTCCA |
| MRG15 | GAGAGCAGAGTACTCAAATACGTG / CTGCCACCATCTCCATTTCCAG |
| MRGX | GGGAAATCACTAGGAGTTCTTGGAAG/TGTGGACCAGCTGTCTTCTTTC |
| MRGBP | CCATCTGAGCACCATGTACGAC/ CCCCAAACTCCCTGAAGATGAA |
| RUVBL1 | GAAGACAGAGGTGCTGATGGAGA/ CACATCTCCAGCTTCTACTCGC |
| RUVBL2 | ATCGCCGGCAGTGAAATCTTCT/ CATCTCTGTGGTCTTGAGGGTCA |

**Table S3. Oligos for ChIP-qPCR analysis**

| ChIP Primers | Sequence | Cell models |
| --- | --- | --- |
| HIV-DHS1-F | GGGACTTTCCGCTGGGGAC | J-Lat10.6 |
| HIV-DHS1-R | CCCAGTACAGGCAAAAAGCAGC | J-Lat10.6 |
| HIV-TSS-F | AGTGGCGAGCCCTCAGATG | J-Lat10.6 |
| HIV-TSS-R | AGCAGTGGGTTCCCTAGTTAGC | J-Lat10.6 |
| HIV-TAR-F | TCTCTGGCTAACTAGGGAACC | J-Lat10.6 |
| HIV-TAR-R | AAAGGGTCTGAGGGATCTCTAG | J-Lat10.6 |
| HIV-DHS2-F | CGAACAGGGACTTGAAAGCGAAA | J-Lat10.6 |
| HIV-DHS2-R | CGTACTCACCAGTCGCCGC | J-Lat10.6 |
| HIV-Gag-F | GGTGCGAGAGCGTCAGTATT | J-Lat10.6 |
| HIV-Gag-R | GCTCCCTGCTTGCCCATACT | J-Lat10.6 |
| HIV-Env-F | CAACTCAACTGCTGTTAAATGGCA | J-Lat10.6 |
| HIV-Env-R | CTGGTCCCCTATGGATACGGATA | J-Lat10.6 |
| 367-F | GTACTTCAAGAACTGCTGACATCGA | HIV_GKO_ |
| 367-R | TCTGAGGGCTCGCCACTC | HIV_GKO_ |
| 460-F | AGTGGCGAGCCCTCAGATG | HIV_GKO_ |
| 460-R | AGCAGTGGGTTCCCTAGTTAGC | HIV_GKO_ |
| 567-F | CCACTGCTTAAGCCTCAATAAAGCT | HIV_GKO_ |
| 567-R | TCCACACTGACTAAAAGGGTCTGA | HIV_GKO_ |
| 610-F/TAR-F | AGTGTGTGCCCGTCTGTTGT | HIV_GKO_ |
| 610-R/TAR-R | TTCGCTTTCAAGTCCCTGTT | HIV_GKO_ |
| 698-F | CGAACAGGGACTTGAAAGCGAAA | HIV_GKO_ |
| 698-R | CGTACTCACCAGTCGCCGC | HIV_GKO_ |
| 944-F | GCAAGCAGGGAGCTAGAACG | HIV_GKO_ |
| 944-R | GGATGGTTGTAGCTGTCCCAGT | HIV_GKO_ |
| 1959-F | GCAATGAGCCAAGTAACAAATCCA | HIV_GKO_ |
| 1959-R | CCTTTTTCCTAGGGGCCCTGC | HIV_GKO_ |
| 4745-F | CTACAATCCCCAAAGTCAAGGAGT | HIV_GKO_ |
| 4545-R | GTCTACTATTCTTTCCCCTGCACT | HIV_GKO_ |
| 8411-F | GGCAGGGATATTCACCATTATCGT | HIV_GKO_ |
| 8411-R | GGTGGTAGCTGAAGAGGCACA | HIV_GKO_ |
| ENO1-TSS-F | GAATGAGTGACGGCTCTCCCGA | J-Lat10.6&HIV_GKO_ |
| ENO1-TSS-R | GCCACTGGGTCTCGTCGCCTA | J-Lat10.6&HIV_GKO_ |
| ENO1-ORF-F | CAAAGCTGGTGCCGTTGAGAA | J-Lat10.6&HIV_GKO_ |
| ENO1-ORF-R | ﻿CCAACTCCTTGCTTGGGAAGT | J-Lat10.6&HIV_GKO_ |
| CA6-TSS-F | CAGCTGGGCAGATCAATTGTA | J-Lat10.6&HIV_GKO_ |
| CA6-TSS-R | GAAGGGCACTTGTCCTGTTGTAA | J-Lat10.6&HIV_GKO_ |

**Table S4. Antibodies used in this study.**

| Antibody | Catalog | Manufacture | Usage |
| --- | --- | --- | --- |
| EP400 | A300-541A | BETHYL | WB, ChIP |
| EP400 | AB70301 | Abcam | WB, IP |
| TRRAP | ab227590 | Abcam | WB |
| BAF250 | PA5-85568 | Thermofisher | WB |
| DMAP1 | 10411-1-AP | Thermofisher | WB, ChIP, IP |
| RNAPII | 05-623 | Millipore | WB, ChIP, IP |
| RNAPII | 61801 | Activ motif | ChIP |
| RNAPII-Ser2p | 61083 | Activ motif | ChIP |
| RNAPII-Ser5p | 61805 | Activ motif | ChIP |
| Tat | MA1-71509 | ThermoFisher | WB, ChIP |
| CCNT1 | 20992-1-AP | Proteintech | WB |
| Tat | ab43014 | Abcam | WB |
| GAS41 | sc-393708 | Santa crutz | WB |
| KAT5 | 10827-1-AP | Proteintech | WB |
| RUVBL1 | ab226001 | Abcam | WB |
| MBTD1 | 26098-1-AP | Proteintech | WB |
| BRD4 | ABE1391 | Proteintech | WB |
| Flag | F1804 | Sigma | WB, IP |
| HA | 26183 | Thermofisher | WB, IP |
| His | MA1-135 | Thermofisher | WB, IP |
| GST | sc-138 | Santa crutz | WB |
| Histone H3 | 07-690 | Millipore | Loading control in WB |
| HDAC1 | PA1-860 | Thermofisher | Loading control in WB |
| GAPDH | sc-47724 | Santa crutz | Loading control in WB |
| HIV p24 | MA1-71515 | Thermofisher | WB |
| Anti-Rabbit IgG | NBP3-11668 | Novus | 2^nd^ antibody in WB |
| Anti-mouse IgG | NBP3-11661 | Novus | 2^nd^ antibody in WB |
| Anti-rat IgG | 7077S | CST | 2^nd^ antibody in WB |
