# Supplementary figures and images for: The human chromatin remodeling complex p400 restricts HIV-1 transcription in a Tat-dependent manner
Source: Nucleic Acids Res. 2025 Dec 18;53(22):gkaf1323. doi: 10.1093/nar/gkaf1323 (PMC12714564; doi:10.1093/nar/gkaf1323)

**Figure S1**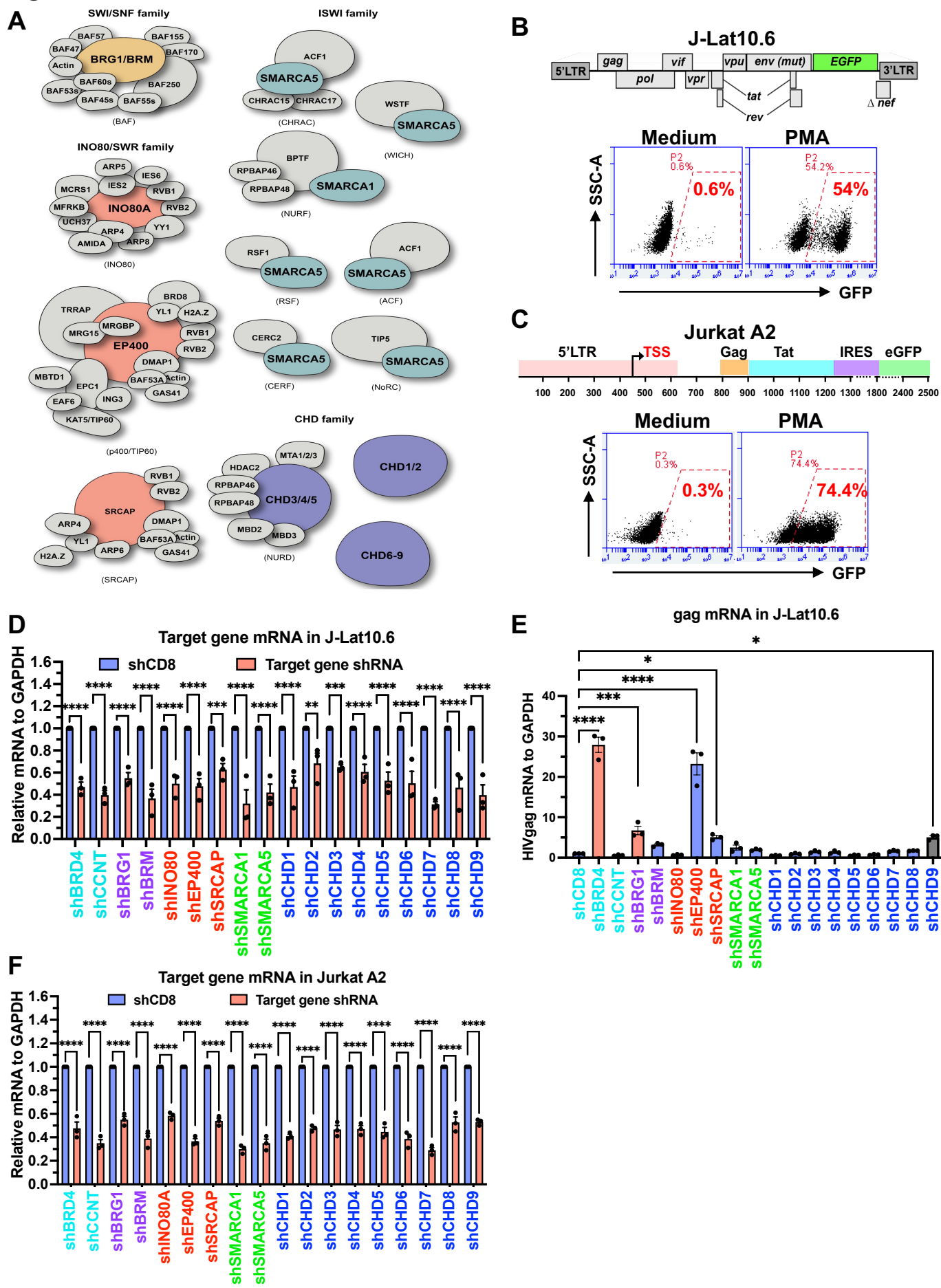

**Figure S2**

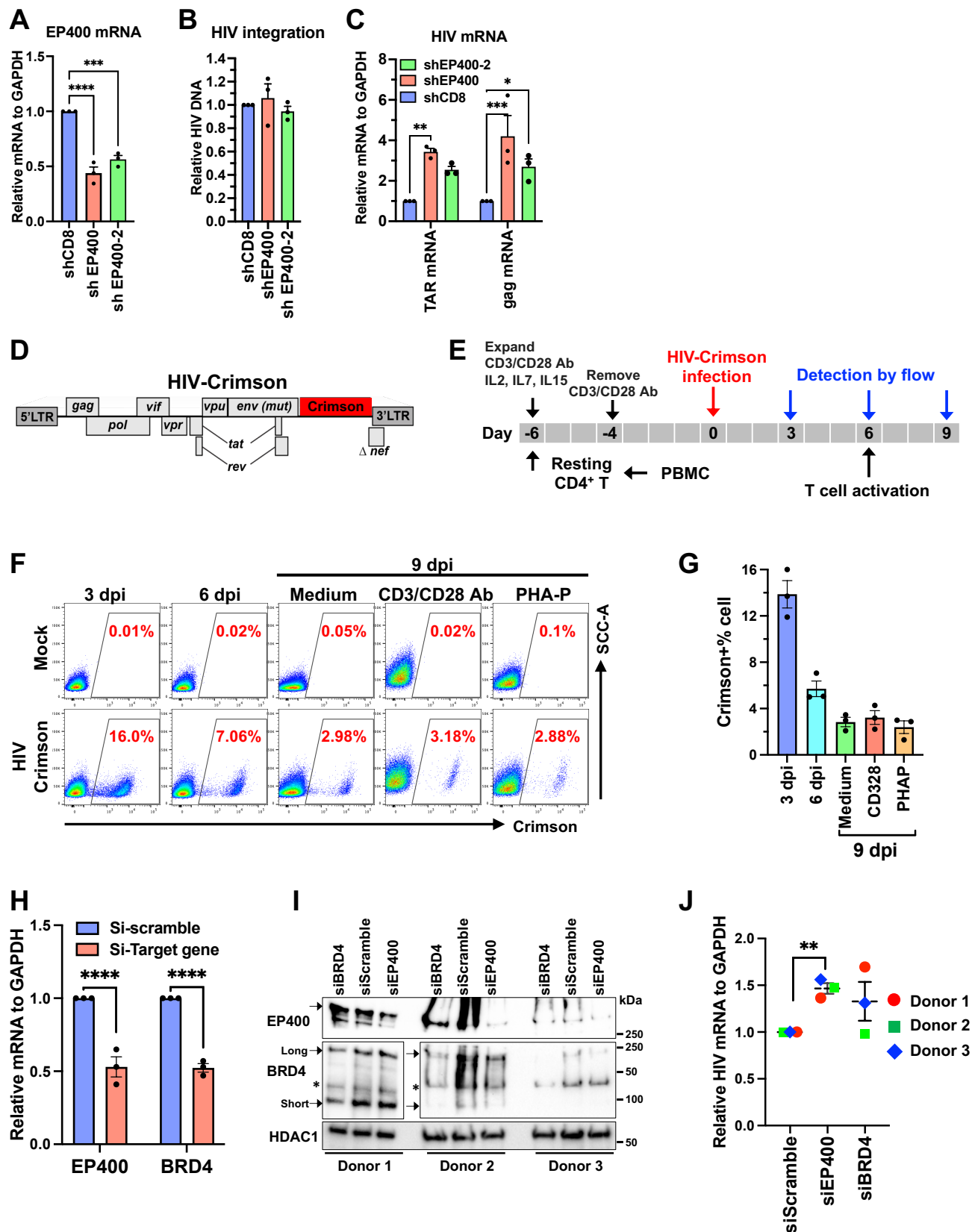

Figure S3

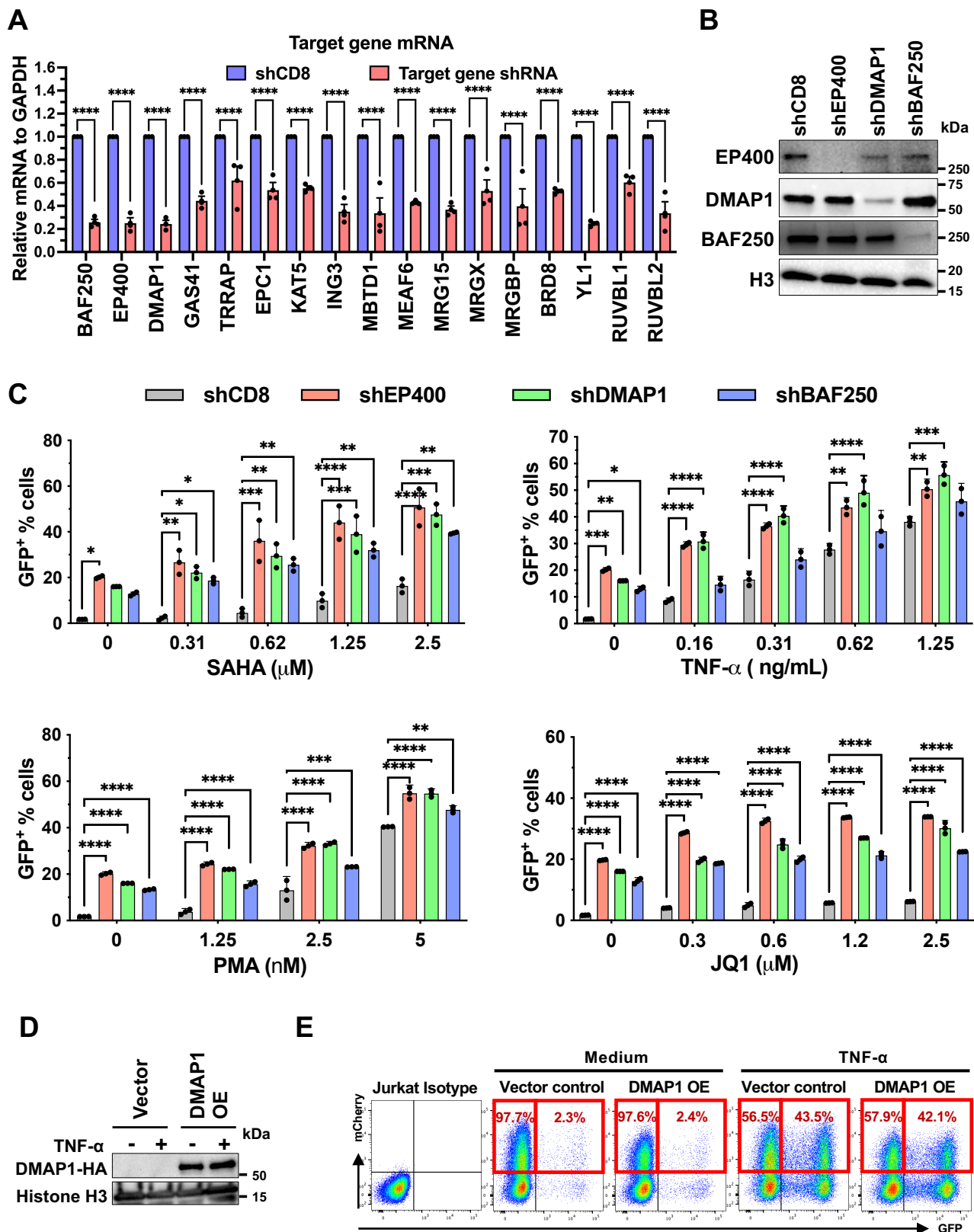

**Figure S4**

**ChIP**

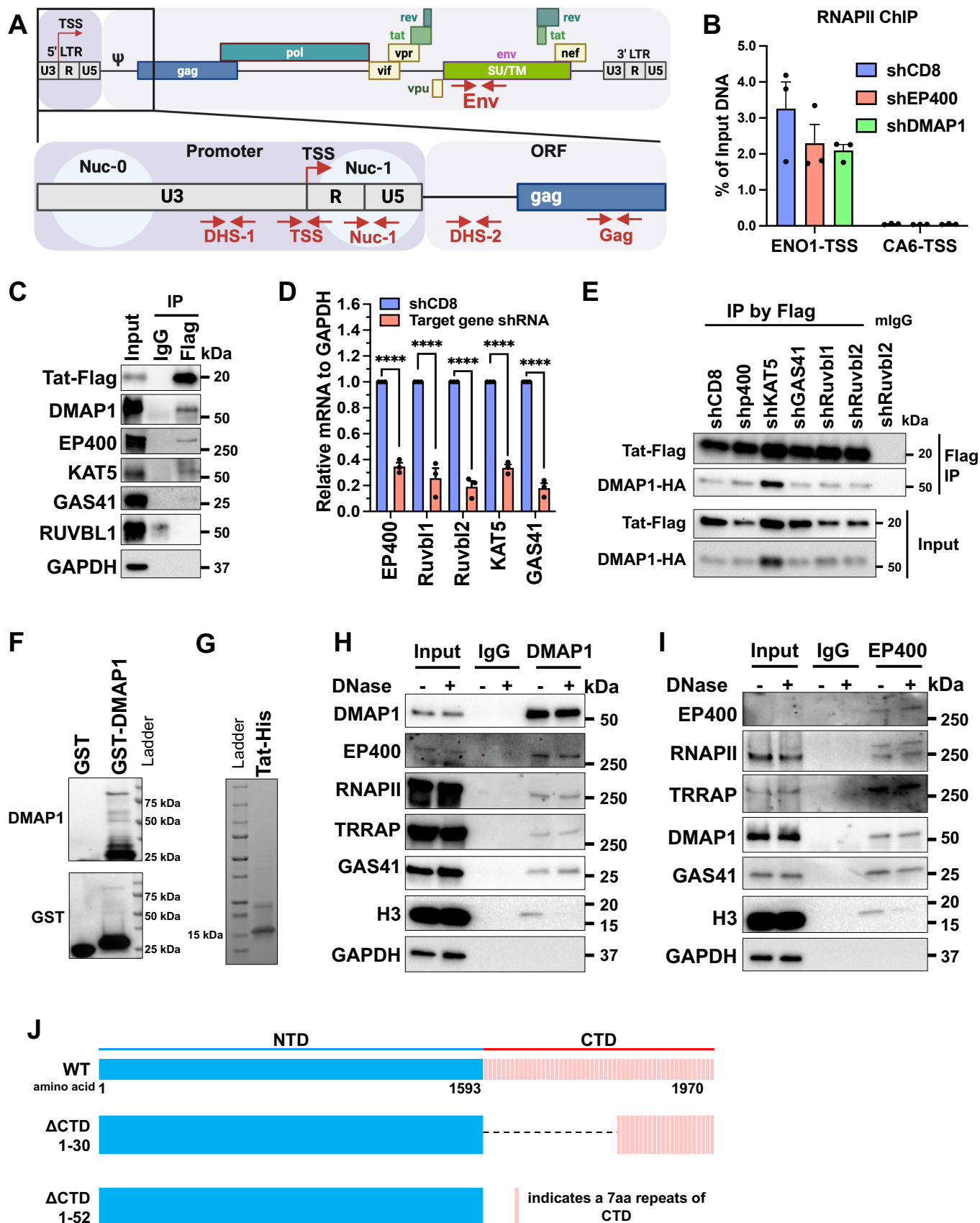

Figure S5

A

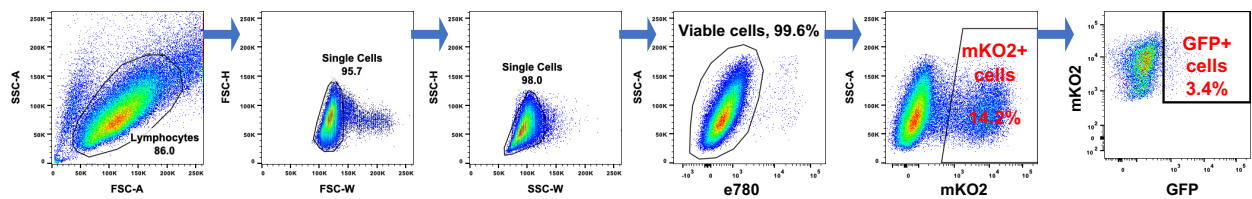

B

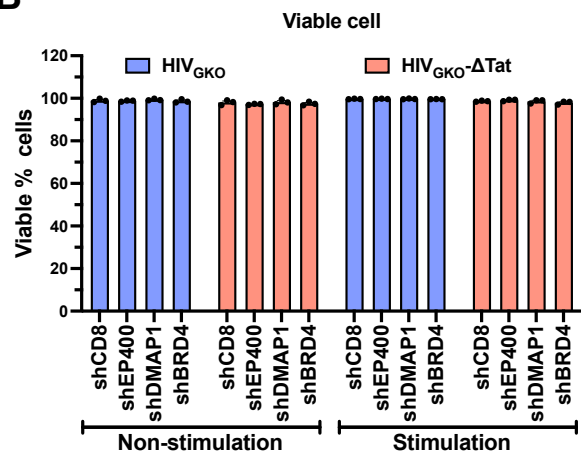

C

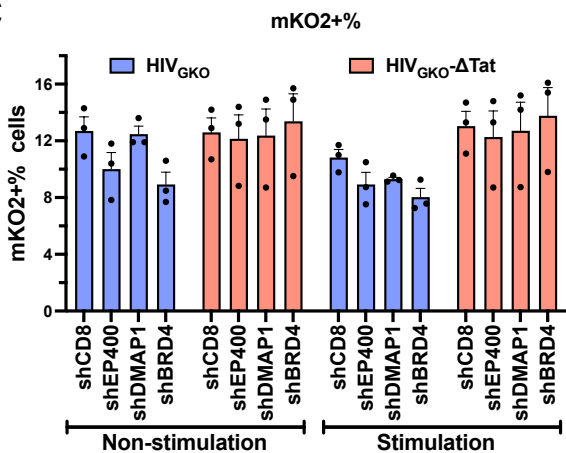

Figure S6

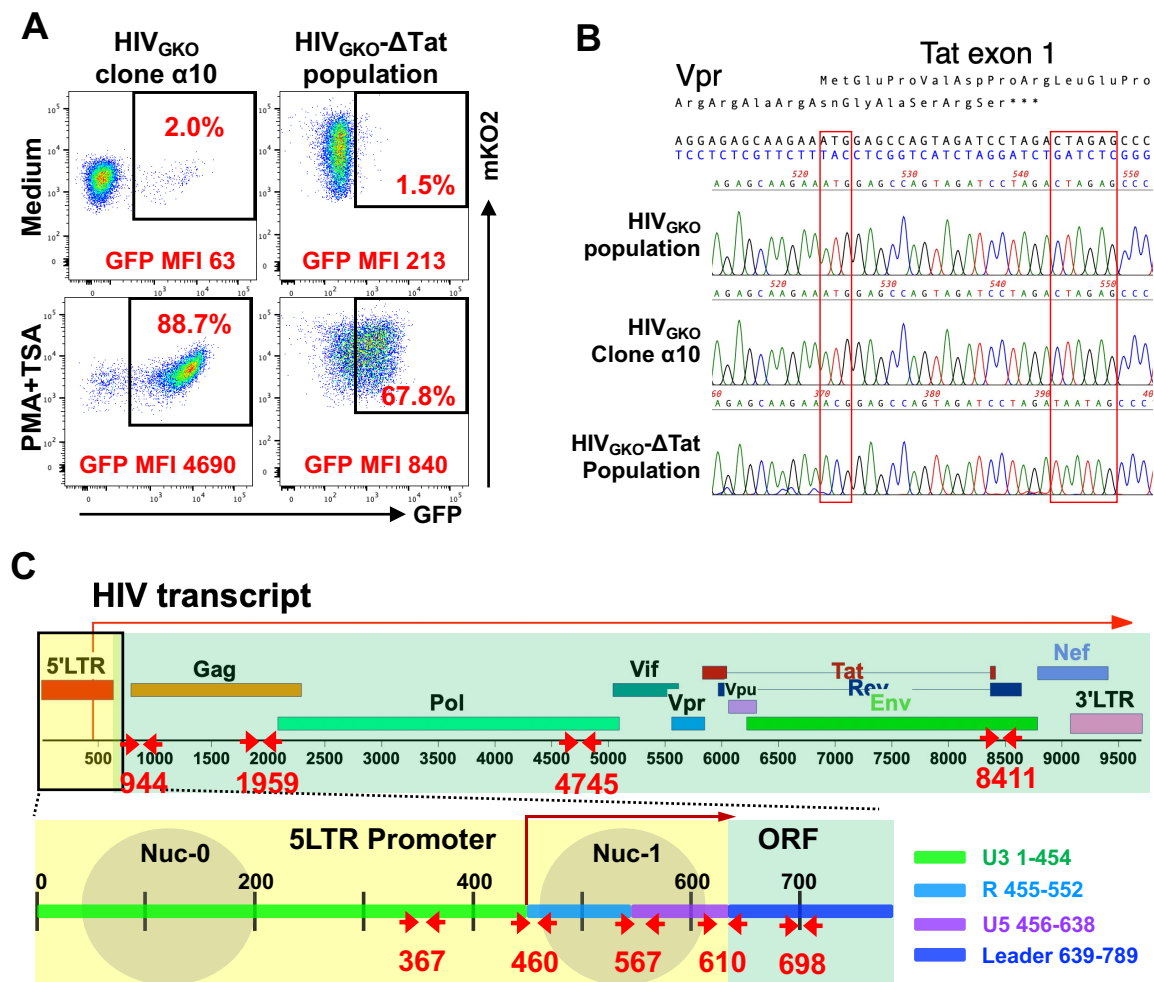

Figure S7

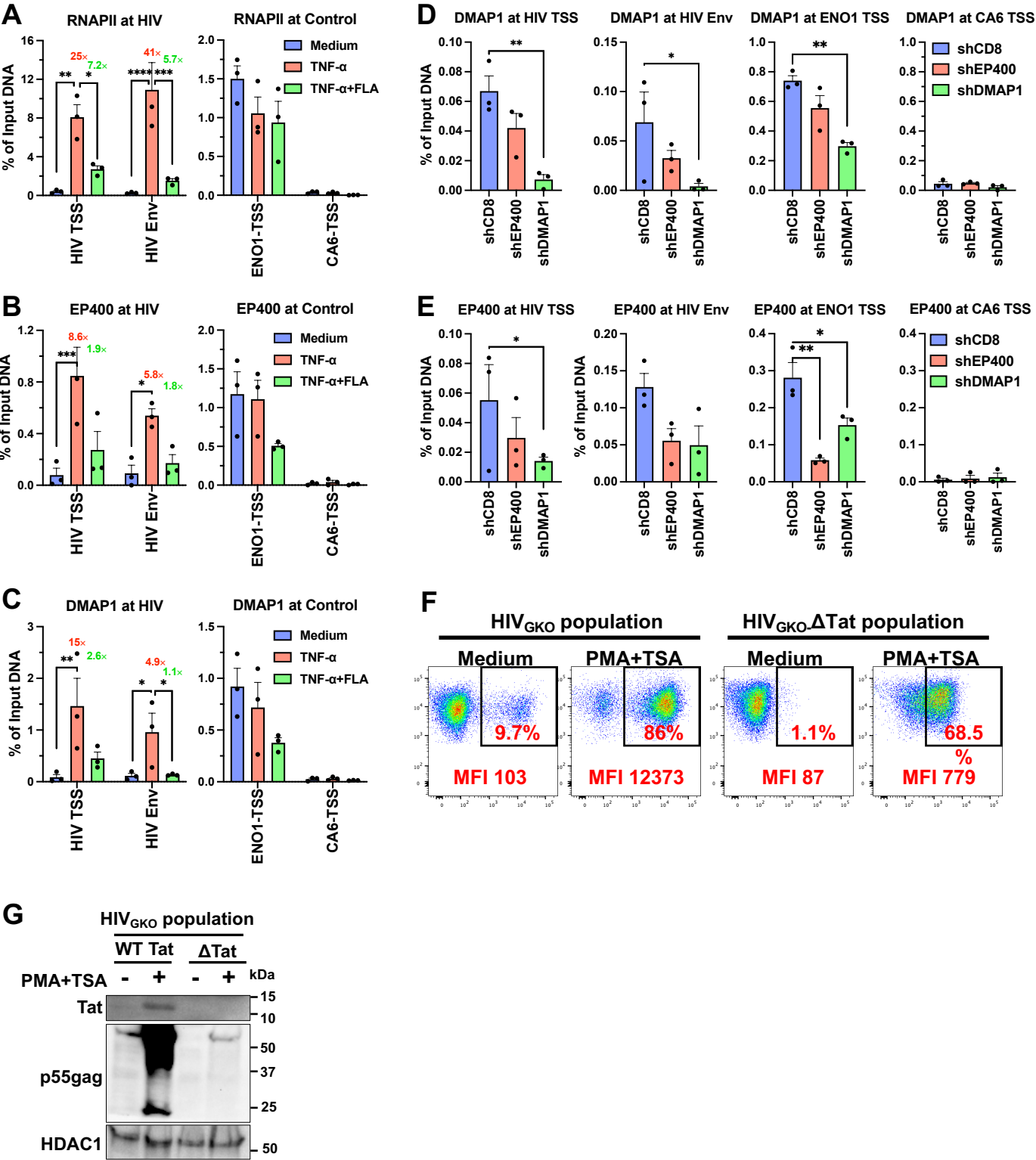

**Figure S8**

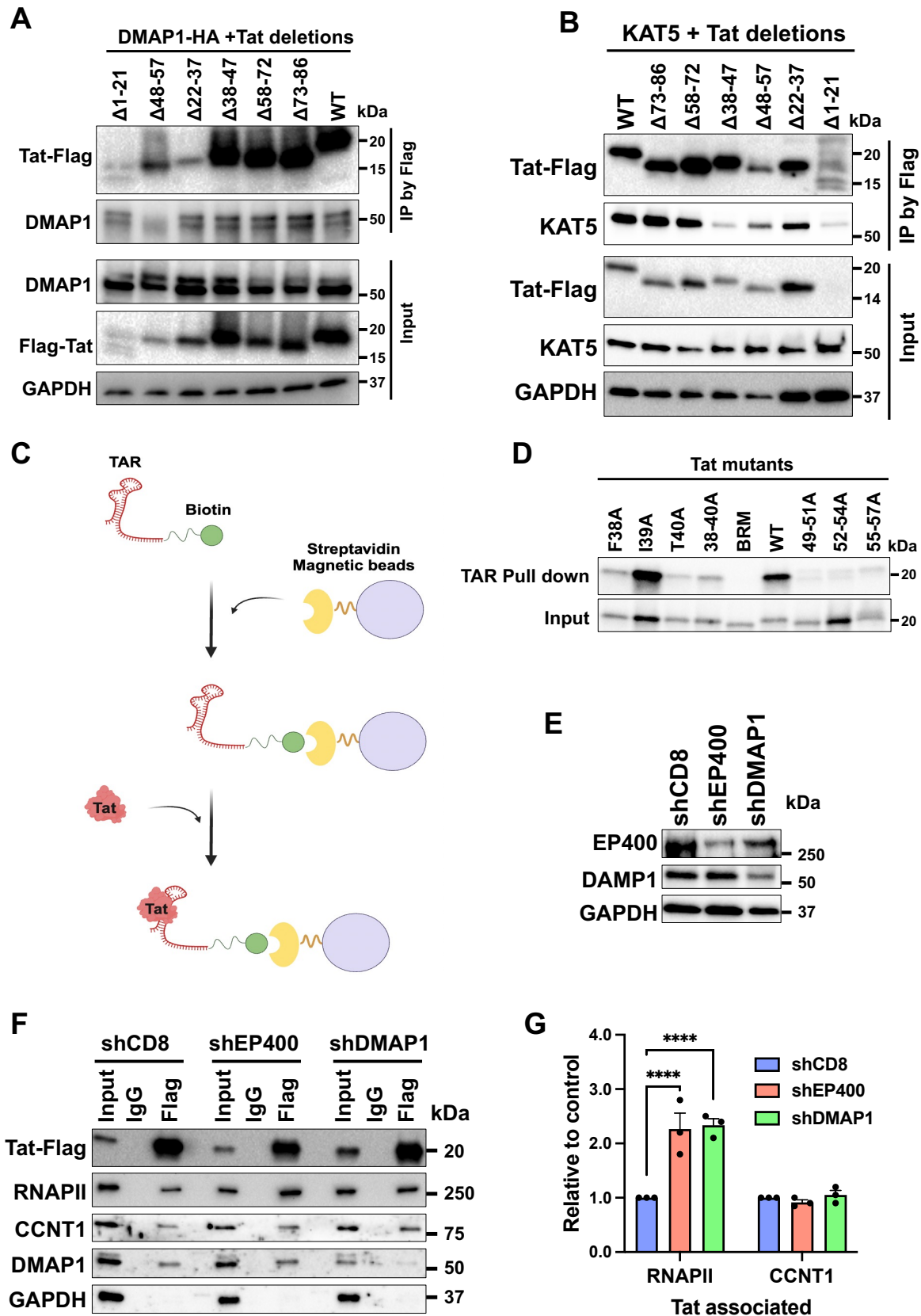

Figure S9

A

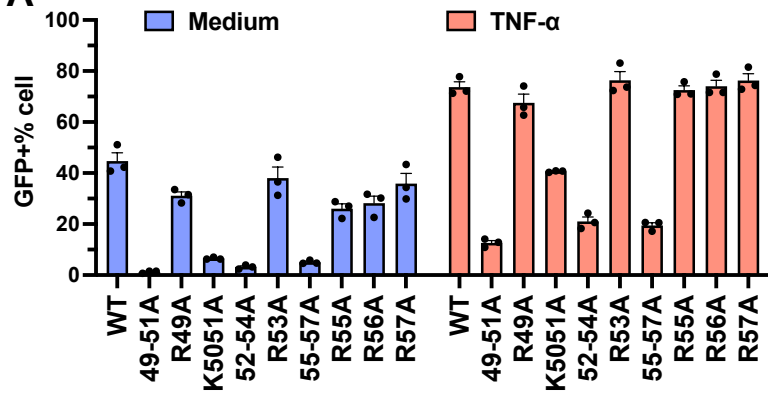

B

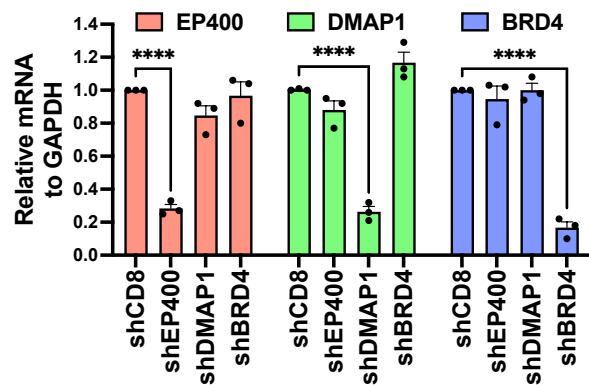

B

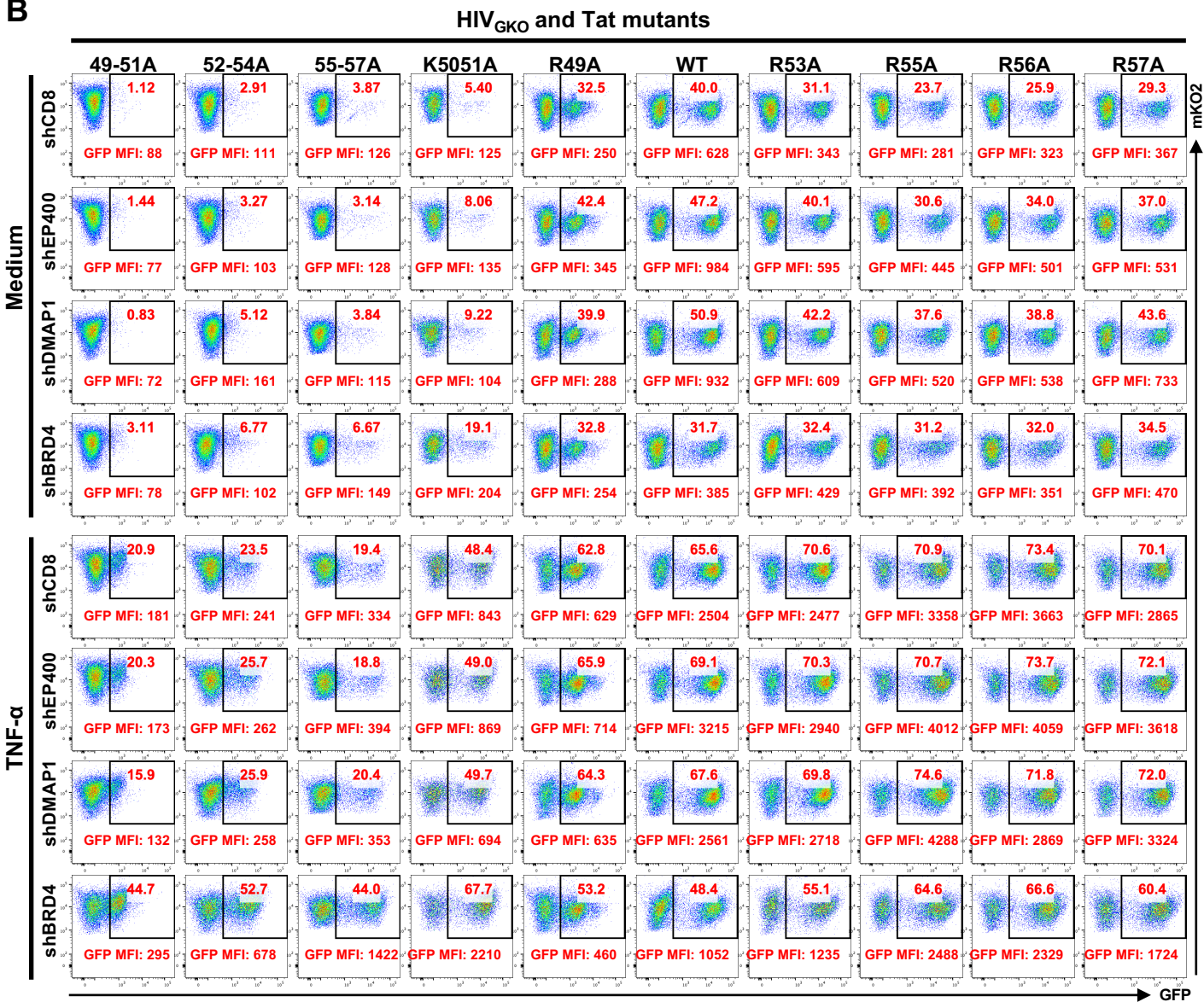

Supplement: gkaf1323_Supplemental_Files [file gkaf1323_supplemental_files.zip › Supplemental figures.pdf]
